# Distinct inhibitory effects on mTOR signaling by ethanol and INK128 in diffuse large B-cell lymphoma

**DOI:** 10.1186/s12964-015-0091-0

**Published:** 2015-03-01

**Authors:** Krystyna Mazan-Mamczarz, Raymond J Peroutka, James J Steinhardt, Moriah Gidoni, Yongqing Zhang, Elin Lehrmann, Ari L Landon, Bojie Dai, Simone Houng, Parameswary A Muniandy, Sol Efroni, Kevin G Becker, Ronald B Gartenhaus

**Affiliations:** Marlene & Stewart Greenebaum Cancer Center, Department of Medicine, University of Maryland, Baltimore, MA 21201 USA; The Mina and Everard Goodman Faculty of Life Sciences, Bar-Ilan University, Ramat-Gan, 5290002 Israel; Gene Expression and Genomics Unit, National Institute of Aging, National Institutes of Health, Baltimore, MD 21224 USA; Veterans Administration Medical Center, Baltimore, MD 21201 USA

**Keywords:** mTOR signaling, Translation, EtOH, INK128, DLBCL, Gene expression

## Abstract

**Background:**

The mechanistic target of rapamycin, (mTOR) kinase plays a pivotal role in controlling critical cellular growth and survival pathways, and its aberrant induction is implicated in cancer pathogenesis. Therefore, suppression of active mTOR signaling has been of great interest to researchers; several mTOR inhibitors have been discovered to date. Ethanol (EtOH), similar to pharmacologic mTOR inhibitors, has been shown to suppress the mTOR signaling pathway, though in a non-catalytic manner. Despite population studies showing that the consumption of EtOH has a protective effect against hematological malignancies, the mechanisms behind EtOH’s modulation of mTOR activity in cells and its downstream consequences are largely unknown. Here we evaluated the effects of EtOH on the mTOR pathway, in comparison to the active-site mTOR inhibitor INK128, and compared translatome analysis of their downstream effects in diffuse large B-cell lymphoma (DLBCL).

**Results:**

Treatment of DLBCL cells with EtOH suppressed mTORC1 complex formation while increasing AKT phosphorylation and mTORC2 complex assembly. INK128 completely abrogated AKT phosphorylation without affecting the structure of mTORC1/2 complexes. Accordingly, EtOH less profoundly suppressed cap-dependent translation and global protein synthesis, compared to a remarkable inhibitory effect of INK128 treatment. Importantly, EtOH treatment induced the formation of stress granules, while INK128 suppressed their formation. Microarray analysis of polysomal RNA revealed that although both agents primarily affected cell growth and survival, EtOH and INK128 regulated the synthesis of mostly distinct genes involved in these processes. Though both EtOH and INK128 inhibited cell cycle, proliferation and autophagy, EtOH, in contrast to INK128, did not induce cell apoptosis.

**Conclusion:**

Given that EtOH, similar to pharmacologic mTOR inhibitors, inhibits mTOR signaling, we systematically explored the effect of EtOH and INK128 on mTOR signal transduction, components of the mTORC1/2 interaction and their downstream effectors in DLBCL malignancy. We found that EtOH partially inhibits mTOR signaling and protein translation, compared to INK128’s complete mTOR inhibition. Translatome analysis of mTOR downstream target genes established that differential inhibition of mTOR by EtOH and INK128 distinctly modulates translation of specific subsets of mRNAs involved in cell growth and survival, leading to differential cellular response and survival.

**Electronic supplementary material:**

The online version of this article (doi:10.1186/s12964-015-0091-0) contains supplementary material, which is available to authorized users.

## Background

The mechanistic target of rapamycin, or mTOR, is a serine/threonine kinase that has been demonstrated to govern a multitude of cellular processes such as survival, growth, metabolism, cell cycle progression, cytoskeletal organization, protein synthesis and autophagy. Functionally, mTOR coordinates the cell’s response to upstream signaling events such as nutrient levels, growth factors, amino acids, oxygen and stress. One or both of the multi-protein mTOR complexes, mTORC1 and mTORC2, convey these signaling events in their activated states. The mTORC1 protein complex contains the unique components of the regulatory associated protein of mTOR (raptor), the proline-rich AKT substrate 40 kDa (PRAS40) and the 12 kDa FK509-binding protein (FKBP12), while mTORC2 exclusively contains the rapamycin-insensitive companion of mTOR (rictor) and the mammalian stress-activated MAP kinase-interacting protein 1 (mSin1) (reviewed by Laplante and Sabatini) [1]. Protein synthesis is the downstream signaling process most frequently studied under the control mTORC1. mTORC1 promotes protein synthesis through the phosphorylation of eukaryotic translation initiation factor 4E (eIF4E) binding protein 1 (4E-BP1) and S6 kinase 1 (S6K1 or p70S6K) (reviewed by Ma and Blenis) [2]. Phosphorylation of 4E-BP1 results in its release of binding to, and inhibition of, eIF4E. eIF4E is then free to form the eIF4F complex and participate in cap-dependent translation. Phosphorylation of p70S6K in turn results in the activation of ribosomal protein S6 (RPS6) and other translational regulators that further enhance translation initiation and elongation. Additionally, active mTORC1 promotes the expression of rRNA and tRNA via interaction with RNA polymerase I (Pol I) and de-repression of Maf1, respectively [3,4]. The summation of active mTORC1 complex’s activities ultimately results in the upregulation of overall protein synthesis while enhancing cell survival mechanisms.

With respect to mTORC1, there has been considerably much less research devoted to the study of the mTORC2 protein complex. It was initially thought that mTORC2 was insensitive to rapamycin, however studies have since demonstrated that long-term treatment can affect mTORC2 signaling in some cell types. Active mTORC2 modulates pathways such as metabolism, survival, apoptosis and growth through the phosphorylation of effector kinases such as the RAC-alpha serine/threonine-protein kinase (AKT) [1]. While the mTORC2 complex partially activates AKT by phosphorylating Ser473, this event facilitates the action of PDK1, which can further enhance the activity of AKT by phosphorylating the Thr308 residue [5]. The activity of the serum/glucocorticoid-regulated kinase 1 (SGK-1) has been demonstrated to rely solely on the activity of the mTORC1 complex. SGK-1 controls FoxO1 and the tumor suppressor FoxO3, modulating both cellular metabolism and survival, respectively [1].

Given the potential power of mTOR signaling with regard to cellular processes, the pathway has been an attractive therapeutic target for various maladies including cancer, type II diabetes, obesity and neurodegeneration. Dysregulation of mTOR signaling in cancer has been observed to result from both aberrant upstream and downstream signaling pathways. Recent studies indicate that mTOR-mediated dysregulation of translation may play a critical role in the development, maintenance and progression of certain malignancies [1]. To date, several rapamycin analogs (rapalogs) have been developed and tested in the clinic, however they have shown limited efficacy. The presence of negative feedback loops, originating from mTOR1 signal transduction, has been proposed as limiting with respect to the rapalogs [1]. Another proposed limitation of rapalog treatment is the partial inhibition of 4E-BP1 [6], which still allows for translation and pro-proliferative/anti-apoptotic signaling in tumors. More recently, two mTOR kinase domain inhibitors (TORKinibs; AZD8055 and INK128) targeting both members of the mTOR complex (mTORC1 and mTORC2), have been developed and are currently being evaluated in several clinical trials [6,7]. Initial results have shown catalytic inhibitors having single-agent activity apparently higher than that seen with rapalogs. Clinical responses to TORKinibs have thus far been seen in patients with non-small cell lung cancer, hepatocellular carcinoma, and estrogen receptor positive breast cancer, with potential utility in diffuse large B-cell lymphoma [8-11].

Together with the rapalogs and recently developed catalytic inhibitors, EtOH has been reported to modulate mTOR signaling and protein synthesis [12-16], yet the mechanisms behind its impact on mTOR component activity (mTORC1 and mTORC2) and its downstream consequences are still under investigation. Interestingly, multiple population studies have shown a decreased incidence of hematological malignancies including leukemia, myeloma, non-Hodgkin lymphoma, and Hodgkin disease [17-21]. Notwithstanding several studies performed on muscle tissue [12,13] or brain cells [22-25], there are very limited studies of EtOH impact on human neoplastic cells [14].

Given that both EtOH and TORKinibs inhibit mTOR signaling, we attempted to systematically compare the effect of EtOH and INK128 on mTOR signal transduction and interaction between components of the mTORC1/2 complexes in diffuse large B-cell lymphoma (DLBCL) malignancy. We used SUDHL-2 cells, representing an aggressive activated B-cell-like (ABC) DLBCL, and SUDHL-4 cells representing germinal center B cell-like (GCB) DLBCL type which usually has a better clinical prognosis. We also evaluated the differences in the mechanisms underlying EtOH and INK128 activity and performed head to head genome-wide characterization of their downstream translationally controlled genes. We found that, in contrast to INK128, which induced complete inhibition of mTORC1 and mTORC2 activity, EtOH decreased mTORC1 activity and complex formation, while concurrently activating AKT phosphorylation and mTORC2 assembly. Consequently, EtOH-triggered suppression of cap-dependent translation and global protein synthesis was less profound as compared to the inhibitory effect of INK128. Importantly, EtOH elicited incomplete inhibition of mTOR signaling and translation while inducing stress granule (SG) assembly, whereas INK128 induced full mTOR inactivation and impaired SG formation. Furthermore, microarray analysis of polysomal RNA revealed that EtOH and INK128, through differential inhibition of the mTOR pathway, distinctly modulated the DLBCL translatome. While both agents primarily affect a group of functionally related mRNAs, which are involved in cell growth and survival, EtOH and INK128 differentially affect the synthesis of distinct subsets of proteins involved in these processes. Finally, both EtOH and INK128 inhibited cell cycle and proliferation, and induced autophagy; EtOH, however, did not induce cell apoptosis. In summary, we provide evidence that EtOH partially inhibits mTOR activity as compared to full inhibition of mTORC1/mTORC2 by INK128 in DLBCL, which results in the differential regulation of downstream mTOR targets and consequent cellular responses.

## Results

### EtOH and INK128 differentially affect mTORC1/2 function in DLBCL cell lines

The disruption of mTOR complexes by different pharmacological and genetic approaches results in varied effects with regard to AKT phosphorylation, protein translation, cell growth and proliferation. EtOH was reported to inhibit mTORC1 signaling and decrease protein synthesis [14,15,22,24]. INK128 (MLN0128) was identified as a highly specific ATP competitive inhibitor of mTOR [26]. Although EtOH and INK128 are very different compounds, they both affect mTOR signaling pathway components; we therefore set out to evaluate the mechanisms underlying their activity by comparing both mTOR inhibitors in parallel. For EtOH treatment, we used a range of concentrations (20, 40, 100 mM), which were in agreement with previous research [12-14,23,25]. Following INK128 dose response studies (Additional file 1: Figure S1), 40 and 200 nM concentrations were determined to be optimal for obtaining equipotent inhibitory effects on mTOR signaling with regard to EtOH treatment. Since there is no evidence for catalytic inhibition of mTOR by EtOH, the prototypical allosteric inhibitor, rapamycin, was used for extensive comparisons.

**Figure 1:**
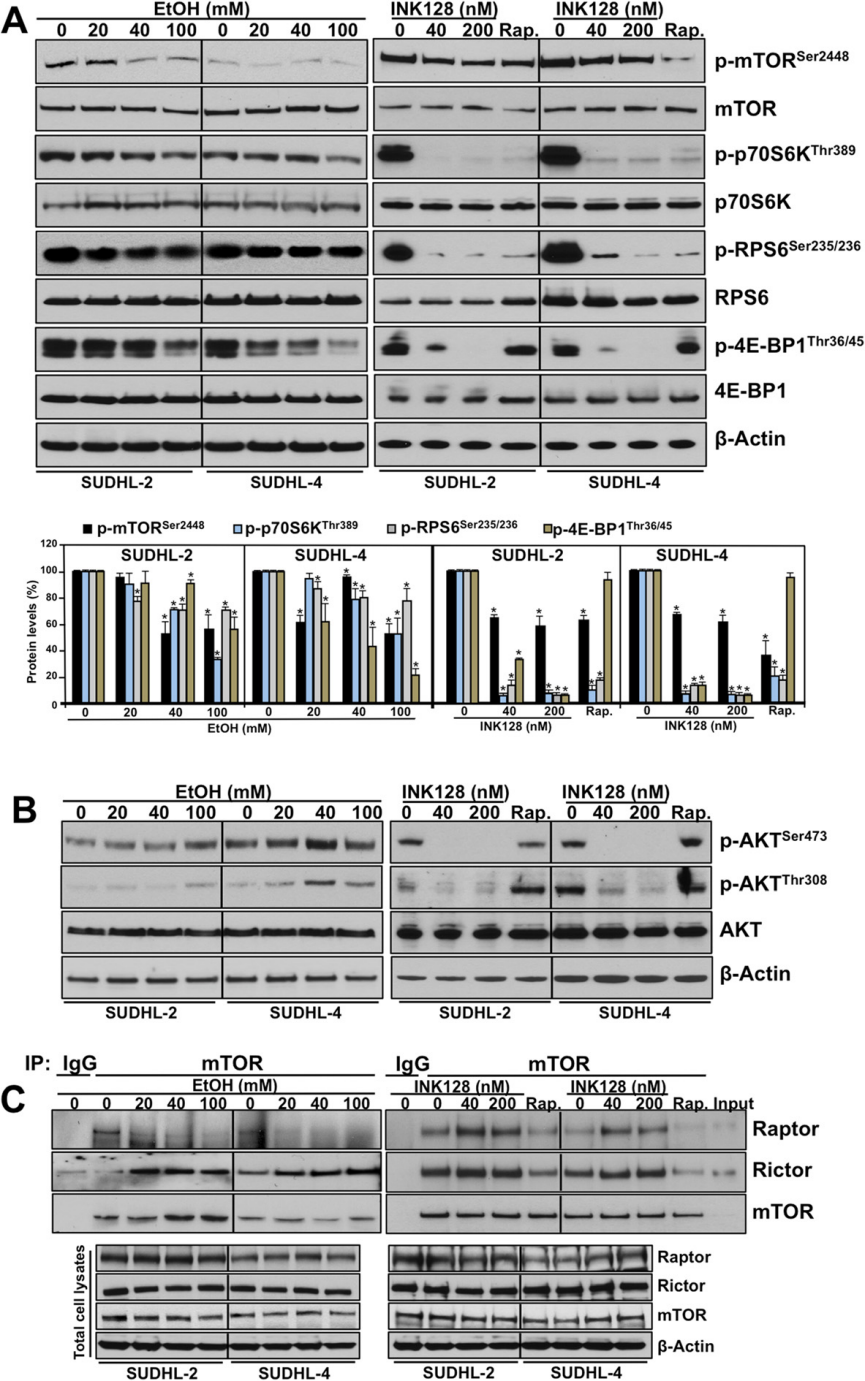
**Effect of EtOH and INK128 on mTORC1/2 activity in DLBCL cells.** SUDHL-2 and SUDHL-4 cells were treated with either EtOH for 24 h, INK128 for 3 h at the concentrations as indicated, or 20 nM rapamycin (Rap.) for 3 h. **(A)** Cell lysates were analyzed by western blotting for phosphorylation states and total levels of indicated proteins. Quantification of the signals is expressed as the percentage of the signal intensity relative to the control group in each cell line. Graphs present the mean and standard deviation from two to five independent experiments. *p ≤ 0.05. **(B)** The levels of phosphorylated AKT at Ser473 and Thr308, and total AKT levels were assessed. **(C)** IP assays, using either IgG or anti-mTOR antibodies, were performed to analyze association of mTOR with raptor and rictor. Immunoprecipitates and total cell lysates were analyzed by immunoblotting (5 ug input). β-Actin served as a loading control. Data are representative of at least three independent experiments.

Exposure to either EtOH or INK128 inhibited the mTORC1 signaling pathway, as indicated by a dose-dependent reduction in the phosphorylation of mTOR on Ser2448 and its downstream molecules in both DLBCL cell lines (SUDHL-2 and SUDHL-4): p70S6K on Thr389, a direct target of mTORC1; RPS6 on Ser235/236, a substrate of p70S6K; and 4E-BP1 on Thr36/45 (Figure 1A and Additional file 1: Figure S1). Similar results were obtained when cells were treated with rapamycin. In agreement with other reports, rapamycin did not affect 4E-BP1 phosphorylation on Thr36/45 sites [27]. To test the effect of both inhibitors on the mTORC2 pathway, we evaluated AKT phosphorylation. EtOH exposure (like rapamycin) either did not change or, at higher concentrations, increased AKT phosphorylation on both Ser473 and Thr308, suggesting that a stronger dose of EtOH might stimulate mTORC2 activity (Figure 1B). In contrast, treatment with INK128 resulted in a complete loss of AKT phosphorylation on Ser473 and a strong decrease in phosphorylation of Thr308 in both doses, confirming INK128’s inhibitory effect on both mTORC1 and mTORC2 complexes. Consistent results were obtained in experiments performed in several B-lymphoblastoid cell lines derived from a healthy patient (Additional file 2: Figure S2), suggesting that both lymphoma cells and normal lymphocytes respond in a similar way to EtOH and INK128 with respect to mTOR activity.

Subsequently, we examined the influence of EtOH and INK128 on protein-protein interactions of the individual mTORC1 and mTORC2 complex members. To this aim, extracts from cells untreated or treated with EtOH, INK128 or rapamycin were immunoprecipitated with antibodies specific to mTOR and then immunoblotted with raptor (component of mTORC1 complex) or rictor (component of mTORC2 complex) antibodies (Figure 1C). EtOH exposure decreased the relative abundance of the raptor-mTOR complex while the mTOR-rictor interaction increased, confirming EtOH’s inhibition of mTORC1 and activation of mTORC2 pathways. Interestingly, despite the strong inhibition of mTOR signaling by INK128 treatment, there was either no effect or an increase in both mTORC1 or mTORC2 complex formations with respect to raptor and rictor association. In agreement with previous reports [28], rapamycin inhibited assembly of both mTORC1 and mTORC2 in studied lymphoma cells. There were no changes in the amount of total cellular levels of rictor or raptor in these experiments. In summary, these data demonstrate that EtOH suppresses mTORC1 activity and complex formation, while concomitantly activating AKT (at both Thr308 and Ser473) and mTORC2 assembly in a manner similar to the feedback observed in rapamycin treatment. In contrast, INK128 appears to fully inhibit the activity of both mTORC1 and mTORC2 without much affect on the mTORC1/2 complex.

### EtOH and INK128 inhibit cap-dependent translation and global protein synthesis

**Figure 2:**
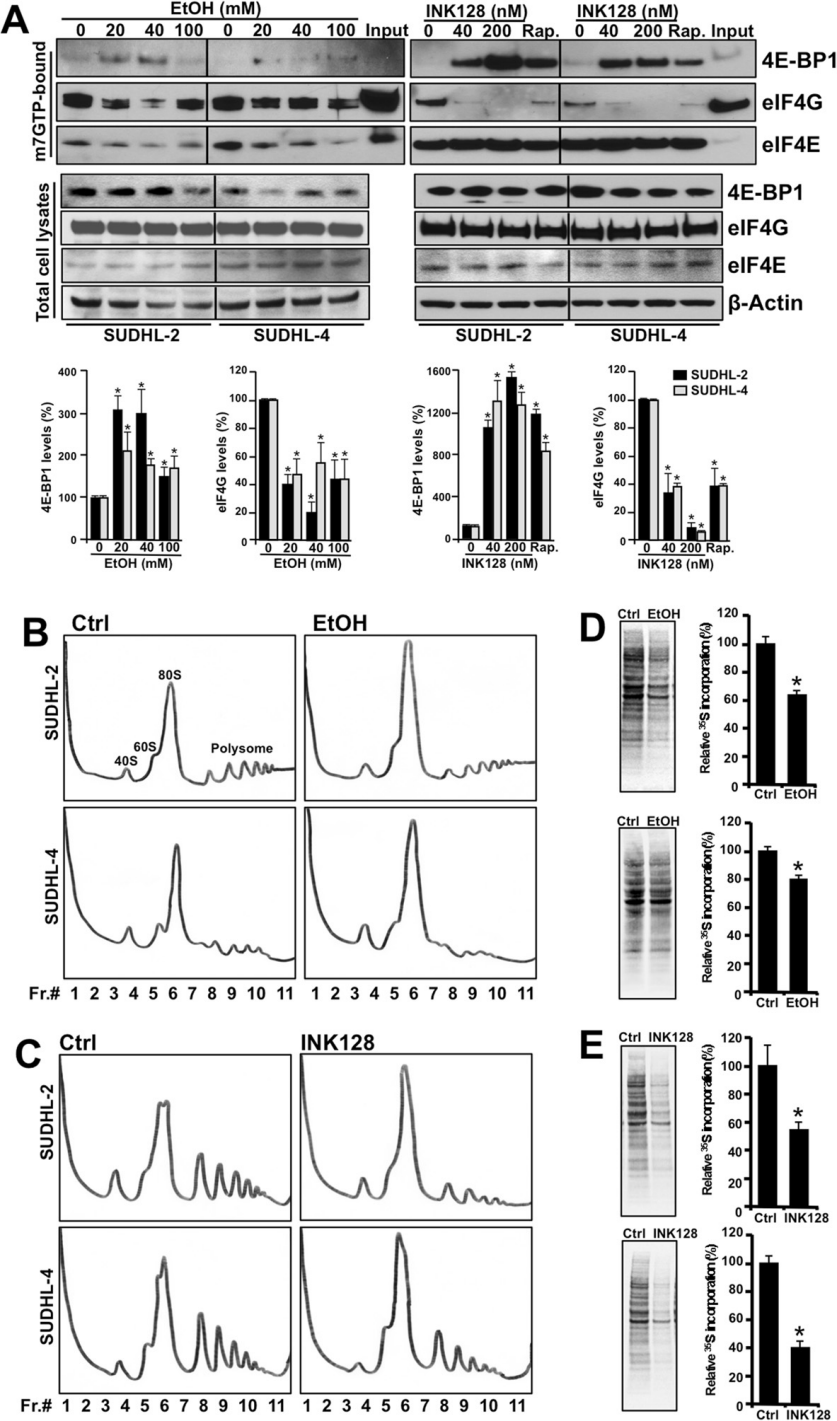
**Effect of EtOH and INK128 on translation of DLBCL cells. (A)** At 48 h after cells were treated with EtOH and 24 h after cells were treated with INK128 or 20 nM rapamycin (Rap.), m7GTP cap analog pull-down reactions were performed. 4E-BP1, eIF4G, and eIF4E protein abundance were analyzed by western blotting along with the total protein levels. 10 μg of protein were used for Input and whole cell lysate blots. **(B,C)** Cells were either untreated (Ctrl) or treated with 20 mM of EtOH for 48 h or 40 nM of INK128 for 24 h. Cell lysates were fractionated through 10–50% linear sucrose gradients (lanes 1 through 11) and the distribution of mRNA associated with ribosomal subunits 40S and 60S, monosomes 80S and polysomes of increasing molecular weight were monitored by 245 nm absorbance. **(D,E)** Cells were treated as described in **(B,C)** and incubated for 20 min with ^35^S-labeled amino acids. Cell lysates were resolved by SDS-PAGE and visualized with a PhosphorImager. Nascent protein synthesis was quantified and graphed as a percentage of signal intensity relative to controls. The data shown are representative of at least three independent experiments. Quantification of the signals is expressed as the percentage of the signal intensity relative to the control group in each cell line. Graphs present the mean and standard deviation from two to five independent assays. *p ≤ 0.05.

Given the role of mTOR in regulating cap-dependent and global translation, we examined the potential for EtOH and INK128 to influence protein synthesis in DLBCL. To test how decreased phosphorylation of 4E-BP1 upon EtOH or INK128 exposure (Figure 1A) modulates 4E-BP1 and eIF4G association with eIF4E, we incubated cell lysates with the 7-methyl-GTP (m7GTP) cap bound to Sepharose beads. Consistent with EtOH’s incomplete inhibition of 4E-BP1 phosphorylation on Thr36/45 (Figure 1A and Additional file 1: Figure S1), EtOH treatment modestly increased association of 4E-BP1 with eIF4E (Figure 2A). As the association of 4E-BP1 and eIF4G with eIF4E is mutually exclusive, the EtOH-triggered accumulation in 4E-BP1-eIF4E complexes appropriately decreased eIF4G-eIF4E interactions in both lymphoma cell lines. INK128 treatment led to almost undetectable association of eIF4G with eIF4E, accompanied by strikingly more abundant 4E-BP1-eIF4E complex formations when compared to the effect of EtOH, while rapamycin had an intermediate effect. No changes were observed in total levels of studied protein.

Since the majority of protein synthesis is cap-dependent, we further assessed the effects of EtOH or INK128 on global mRNA translation. Global changes in protein synthesis were measured using two experimental approaches. First, we investigated the consequence of each treatment on overall mRNA translation by monitoring the distribution of polysome formation. Cytoplasmic extracts from the untreated, EtOH, and INK128 treated cell cultures were fractionated on sucrose gradients and the association of mRNAs with the translational apparatus was monitored by 254 nm absorbance. Although both EtOH and INK128 treated DLBCL cultures displayed a reduction in the content of polysomes when compared with controls, INK128’s effect was more prominent (Figure 2B,C). Second, we assessed the incorporation of ^35^S-labeled amino acids into newly translated protein after a short incubation with L-[^35^S]methionine and L-[^35^S]cysteine. In agreement with the polysomal data, we observed a significant decrease in the rate of global nascent protein synthesis in both EtOH and INK128 treated cells relative to the untreated controls (Figure 2D,E). Overall, these results provide evidence that, whereas both treatments suppressed cap-dependent translation and global protein synthesis, EtOH’s inhibitory effects are less pronounced when compared to INK128.

### EtOH induces while INK128 impairs stress granules formation

**Figure 3:**
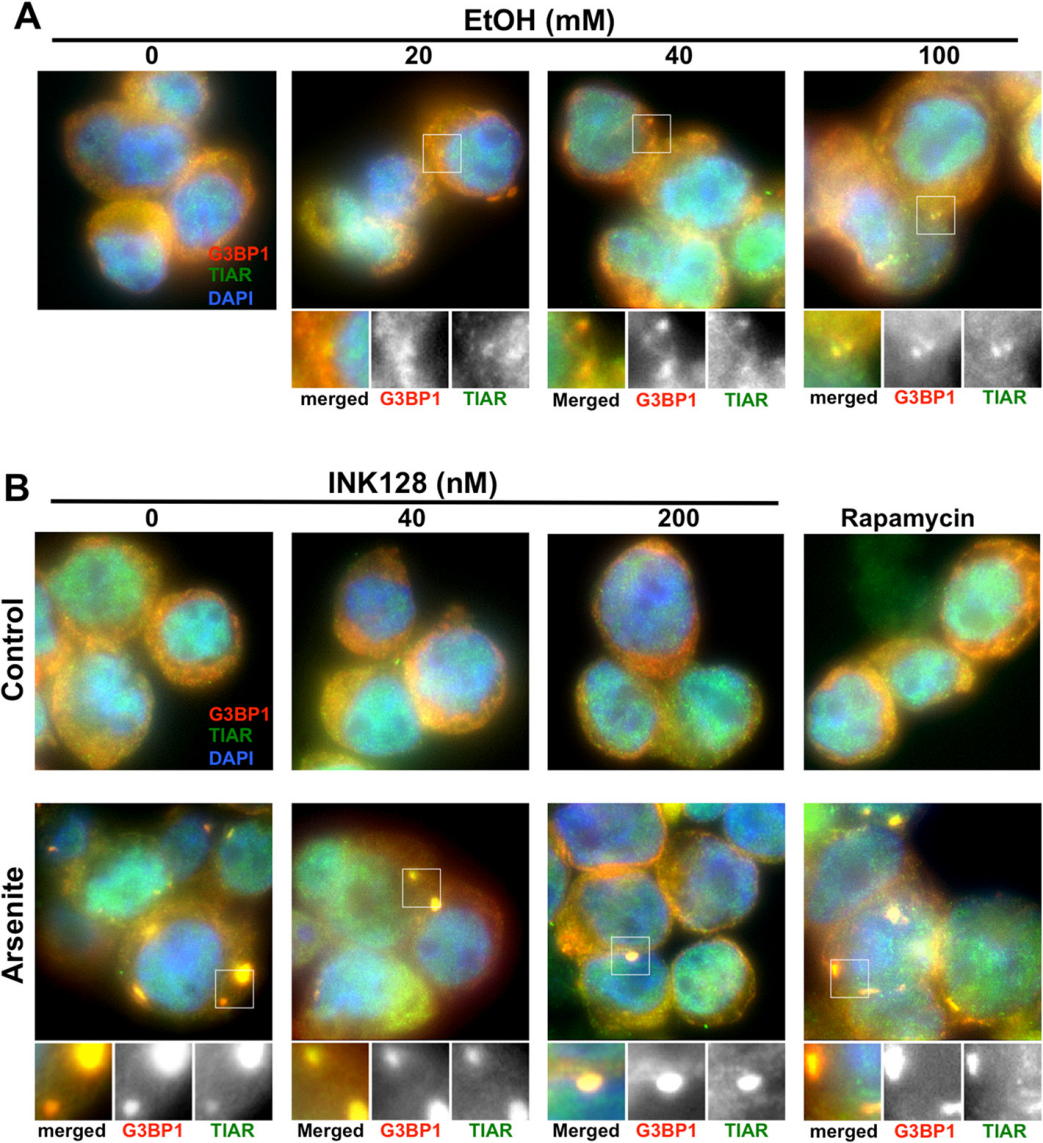
**EtOH induces while INK128 impairs stress granules formation. (A)** SUDHL-2 cells were either left untreated or treated with indicated doses of EtOH, and SG were detected using anti-G3BP1 (red) and anti-TIAR (green) antibodies. DAPI (blue) was used for nuclear staining. **(B)** SUDHL-2 cells were treated with indicated concentrations of INK128 (Control) or preincubated with INK128 for 1 h and then treated with 250 μM arsenite for an additional 1 h (Arsenite). Cells were then analyzed by immunofluorescence as described in **(A)**. Representative fields from three independent experiments are shown. Typical SG are illustrated in enlarged pictures. Pictures were taken using a Nikon TE2000S.

Stress granules (SG) are cytoplasmic foci that accumulate translationally silenced messenger ribonucleo-protein (mRNP) under conditions that inhibit cap-dependent translation initiation [29]. Recent evidence indicates that SG also play a role in inhibition of the mTOR pathway [30-32]. Therefore we investigated the potential for EtOH or INK128 to trigger translational repression through SG formation. SUDHL-2 cells were treated with either EtOH or INK128 and fixed with paraformaldehyde. SG were visualized by immunofluorescence through the co-localization of the classical SG markers G3BP1 and TIAR [33,34]. Corresponding to the EtOH-repressed translation (Figure 2), EtOH treatment efficiently induced SG assembly in a dose dependent manner (Figure 3A). INK128 treatment, however, despite its greater inhibitory effect on mTOR signaling and translation as compared to EtOH (Figure 2), did not cause appearance of SG foci in the SUDHL-2 cells (Figure 3B; *upper panel*). This result and other’s data demonstrating that pharmacological inactivation of the mTORC1-eIF4E pathway may impair SG formation [35] prompted us to wonder whether the dual mTORC1/mTORC2 inhibition by INK128 (Figure 1) resulting in an almost complete eIF4E-eIF4GI complex disruption (Figure 2A) can alter the process of SG formation. In this case, SUDHL-2 cells pretreated with INK128 were next treated with sodium arsenite. As illustrated in Figure 3B (*lower panel*) the number and size of SG assembled upon arsenite treatment declined with increasing dose when cells were pretreated with INK128, suggesting that catalytic inhibition of mTOR by INK128 impairs the SG response. Interestingly, rapamycin treatment neither induced nor disrupted SG formation. Overall, these results demonstrate the previously unreported finding that EtOH treatment, in contrast to both rapamycin and INK128, elicits a moderate inhibition of mTOR signaling and translation while inducing SG assembly, while complete mTOR inactivation, and the translational decline caused by INK128 exposure, result in a failure to form SG.

### EtOH and INK128 exposure distinctly modulate specific gene translation

**Figure 4:**
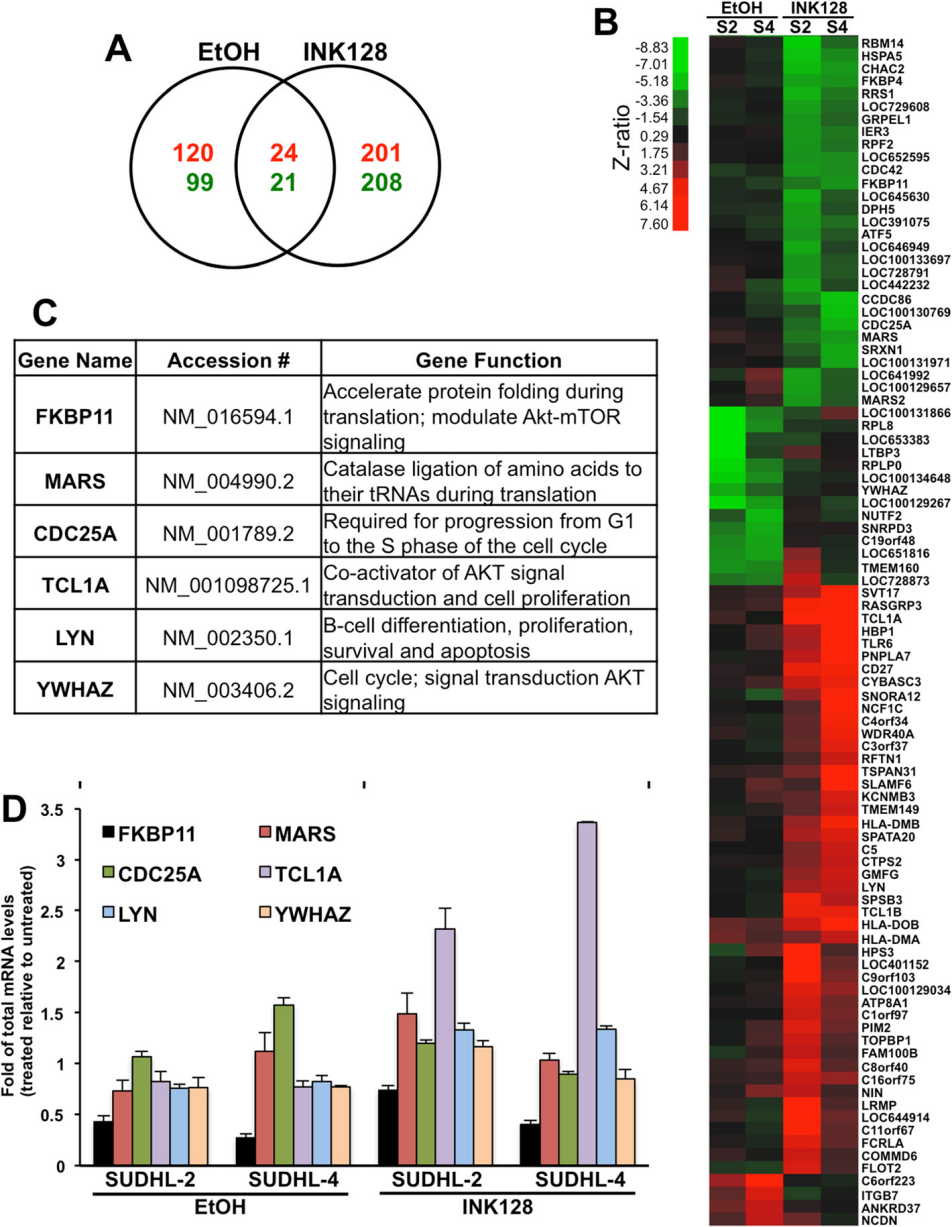
**EtOH and INK128 influence on gene translation in DLBCL cells.** Microarray analysis of translationally active (pooled fractions 9–11) polysomal mRNAs following treatment either with EtOH (20 mM) for 48 h or INK128 (40 nM) for 24 h was performed in three independent replicates. **(A)** Venn diagram comparison of EtOH and INK128 triggered translatome alteration in both SUDHL-2 and SUDHL-4 cell lines; transcripts with significantly (criteria in Material and Methods) increased (red) or decreased (green) translation following treatments when compared to untreated cells. **(B)** The heat map represents top transcripts with the most altered translation induced by EtOH and INK128. S2, SUDHL-2 cells; S4, SUDHL-4 cells. **(C)** Examples of genes showing differences in mRNA translation followed by either EtOH or INK128 exposure. **(D)** Total mRNAs levels of validated genes described in **(C)**, measured by RT-qPCR in cells treated with EtOH or INK128 compared to untreated cells. Graphs represent the mean and standard error of the mean from three repeats of three independent experiments.

In order to better understand the molecular mechanisms wherein EtOH or INK128 trigger global translational repression, we attempted to gain insight into the mRNAs affected by EtOH and INK128 at the translation level. To this end, RNA isolated from translationally active polysomal fractions (pooled fractions 9–11) was subjected to microarray analysis. Venn diagram analysis of the common and specific genes changed within lymphoma cell treatments revealed that EtOH was responsible for modulating, at the translational level, 219 mRNAs (120 mRNAs increased and 99 decreased) as compared with control cells, in both SUDHL-2 and SUDHL-4 cells (Figure 4A). In comparison, INK128 altered the abundance of 409 transcripts (201 increased, 208 decreased) in translationally active polysomal fractions as compared to controls. Only 45 genes were found to be overlapping between both treatments indicating that there is a differential mechanism by which both inhibitors act on mTOR signaling.

**Figure 5:**
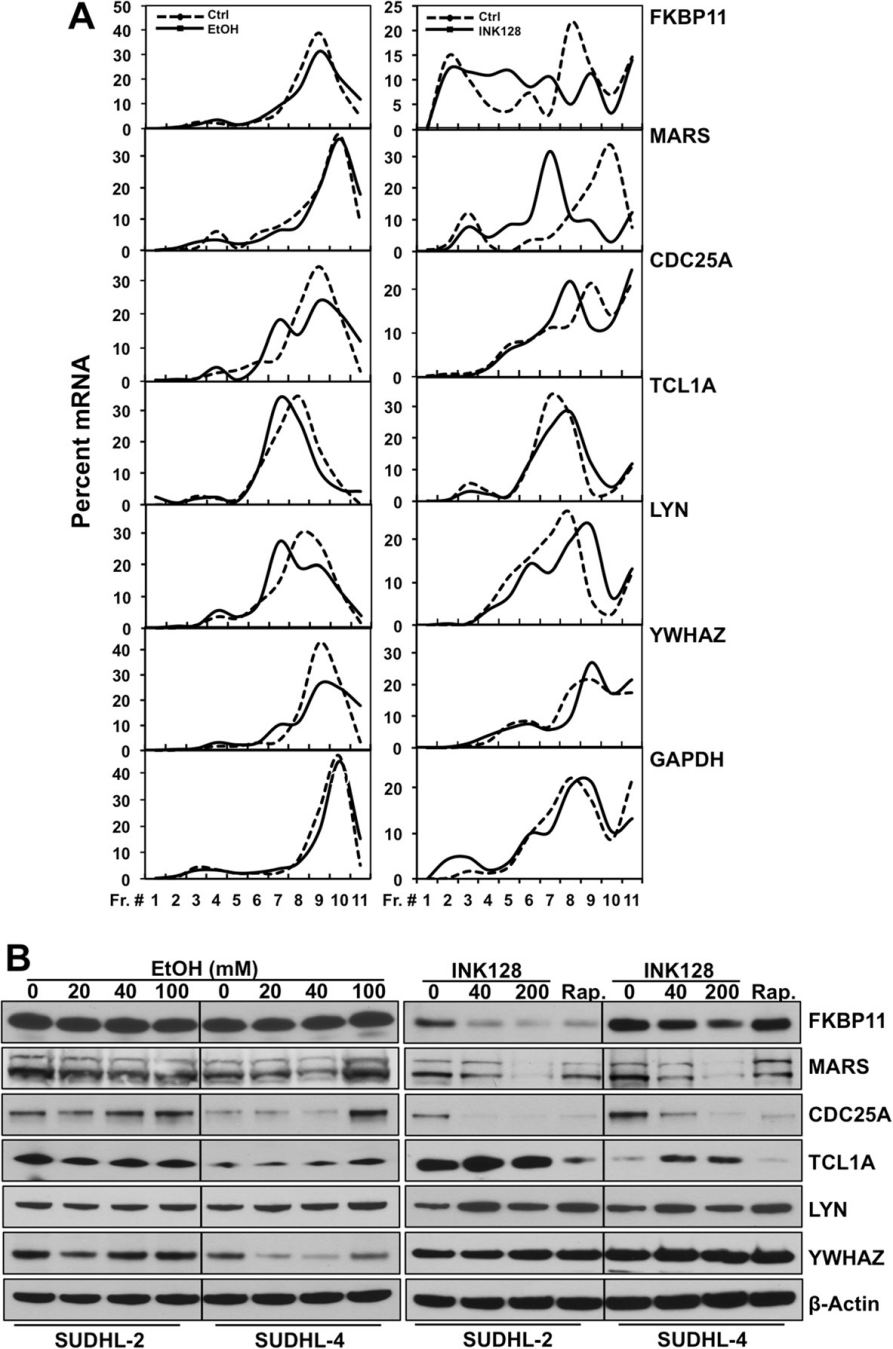
**Validation of microarray data. (A)** SUDHL-2 cells were treated as described in Figure 4. Cell lysates were fractionated through sucrose gradients and EtOH- or INK128-induced changes in translational profile of selected for validation genes (and housekeeping GAPDH mRNA) were monitored by RT-qPCR analysis of RNA from each of 11 fractions. **(B)** Protein levels of validated genes were analyzed by western blotting. β-Actin was used as a loading control. Rap., 20 nM rapamycin. Data are representative of three independent experiments.

Importantly, among the top genes significantly changed consistently in both lymphoma cell lines, we found numerous genes involved in cellular functions, such as AKT/mTOR signaling and protein synthesis (eg. FKBP4, FKBP11, YWHAZ, MARS), cell cycle (eg. CDC25A, CDC42), proliferation and apoptosis (eg. TCL1A, TCL1B, LYN, CD27) (Figure 4B and Additional file 3: Table S1). Therefore, six candidate genes representing each of the pathways above were selected for further validation (Figure 4C). EtOH and INK128 did not alter the total mRNA levels for most of the validated transcripts, with the exception of a ~2 fold decrease observed in FKBP11 mRNA upon EtOH treatment, and a ~3 fold increase in TCL1A mRNA induced by INK128 treatment (Figure 4D). These results indicate possible additional involvement of transcriptional mechanisms in regulation of these two genes. In agreement with the microarray data, EtOH did not modulate or only slightly changed the translational profile of FKBP11, MARS, CDC25A, TCL1A and LYN mRNAs, while INK128 treatment decreased levels of FKBP11, MARS, CDC25A transcripts in the actively translating fractions of the gradient (fractions 9–11) and elevated levels of TCL1A and LYN mRNAs in these fractions, with a subsequent mRNA shift in non-translating (fractions 1–6) and low-translating (fractions 6–8) fractions of the density gradient (Figure 5A). Reverse, we found decreased translation of YWHAZ transcript in EtOH treated groups with no changes upon INK128 treatment as predicted by microarray analysis. No changes were observed in housekeeping GAPDH mRNA used as a loading control. In accordance with the differences obtained in translational profiles, western blot analysis showed concurrently altered protein levels of the six candidate genes (Figure 5B).

To elucidate the potential for functional consequences of EtOH and INK128 treatment, the significantly changed genes were subjected to Ingenuity Pathways Analysis (IPA) and Gene Ontology (GO) analysis. In IPA analysis, genes altered in both treatments were distributed into networks defined by known molecular interactions. For both treatments, the top networks with the highest number of altered genes were molecular functions linked to cell death and survival, cellular development, cellular growth and proliferation, hematological system development and function and tissue morphology (Additional file 4: Table S2). GO analysis identifies biologically functional categories that consist of genes altered upon treatments compared to controls. Not surprisingly, translation and translation-related GO terms encompassed the greatest subset of genes significantly altered with INK128 treatment in both SUDHL-2 and SUDHL-4 lymphoma cells (Additional file 5: Figure S3). These results suggest that although both EtOH and INK128 affect subsets of functionally related mRNAs, which are involved in cell growth and survival, the effect of EtOH treatment on the translation of many of these mRNAs was less profound than INK128.

Altogether, translatome analysis indicated that the majority of those transcripts which were impacted by either treatment pertain to cell growth and survival. In addition, microarray data demonstrated that INK128, affects the synthesis of many proteins involved in the mTOR pathway, translational control and cell proliferation more strongly than EtOH, which is in line with the different impact of these agents on overall protein synthesis rates.

### EtOH and INK128 influence on cell cycle, proliferation and apoptosis

**Figure 6:**
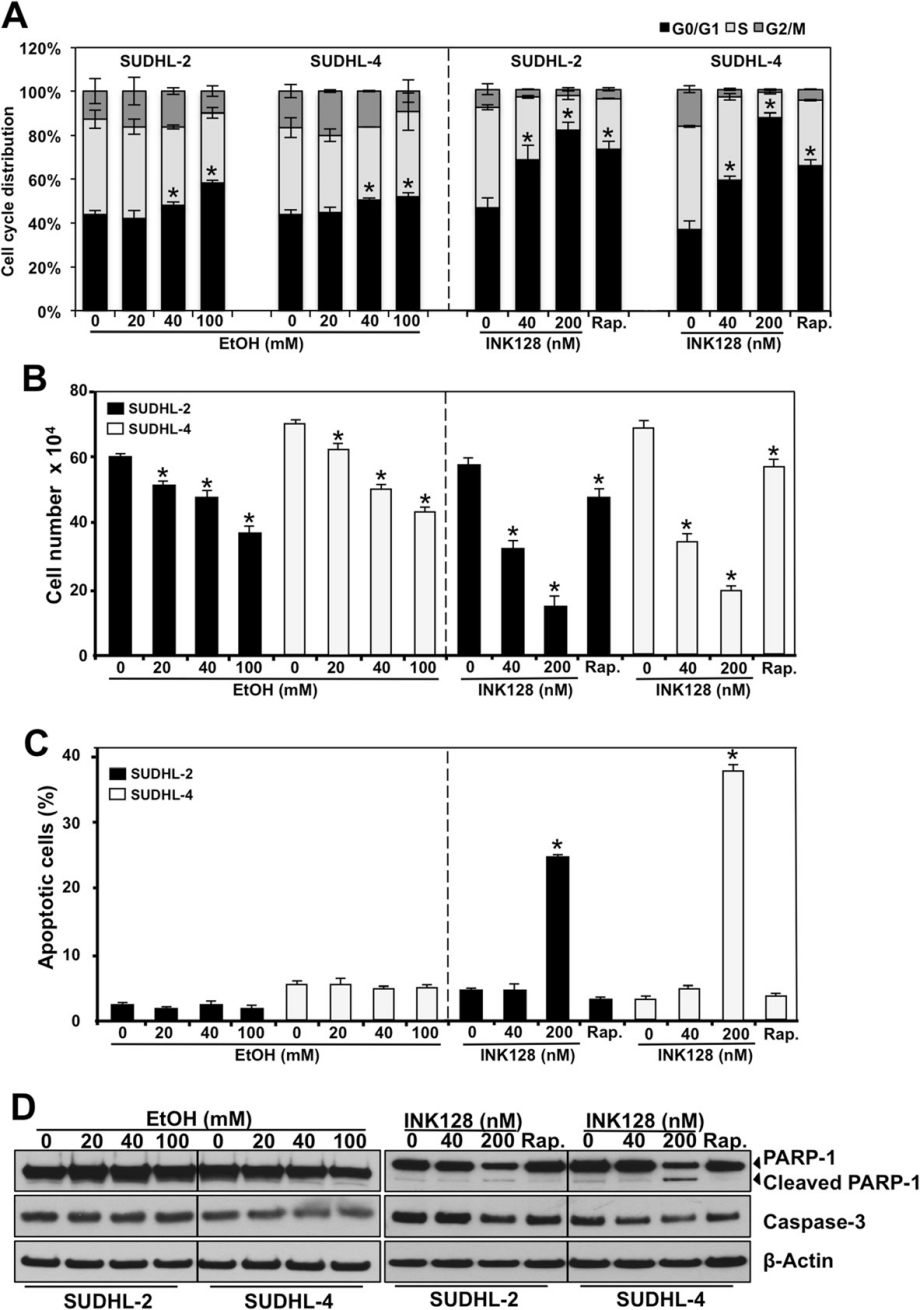
**Influence of EtOH and INK128 on cell cycle,** **proliferation and apoptosis of DLBCL cells.** Cells were treated with indicated doses of EtOH or INK128 as described in Material and Methods. **(A)** At 48 h after treatment, cells were stained with PI and cell cycle distribution was monitored by flow cytometry. **(B)** At 72 h after indicated treatments, cell numbers were counted with a hemocytometer, **(C)** cells were stained with Annexin V and PI, and percent of apoptotic cells was measured by flow cytometry or **(D)** cleavage of PARP-1 and Caspase-3 was analyzed by western blotting. β-Actin served as a loading control. The data are representative of at least three independent repeats. In the graphs **(A)**, **(B)** and **(C)**, the means and SD are shown. *p ≤ 0.05. Rap., 20 nM rapamycin.

Finally, we examined the influence of EtOH and INK128 on cell phenotype. In order to determine how the differences between EtOH and INK128 treatment, along with the observed changes in mTOR signaling and gene expression, impact DLBCL, we examined cell cycle, proliferation and apoptosis. In both cell lines EtOH exposure resulted in only a slight increase in cell cycle arrest, with higher doses (40 and 100 mM) showing a greater percentage of cells in G0/G1 phase (Figure 6A and Additional file 6: Figure S4). INK128 and rapamycin, however, induced stronger cell cycle arrest when compared to EtOH. A dose dependent decrease in cell number was observed with EtOH and INK128 treatment, compared to the untreated cell populations (Figure 6B). Apoptosis was measured by PI/Annexin V staining and confirmed by western blot detection of Caspase-3 and PARP-1 cleavage. Using flow cytometry, no significant evidence of apoptosis was measured for cells treated with EtOH, 40 nM INK128 or rapamycin (Figure 6C and Additional file 7: Figure S5), with no notable protein cleavage detected by western blot (Figure 6D). However, treatment with 200 nM of INK128, resulted in a 20-40% increase in apoptotic cells, as evidenced by PARP-1 cleavage in both SUDHL-2 and SUDHL-4 cell lines. These observations indicate that EtOH is cytostatic, even at its highest dose (100 mM), while increasing the concentration of INK128 from 40 to 200 nM results in cytotoxicity within the two DLBCL cell lines tested.

### EtOH induces autophagy but not apoptosis in DLBCL

**Figure 7:**
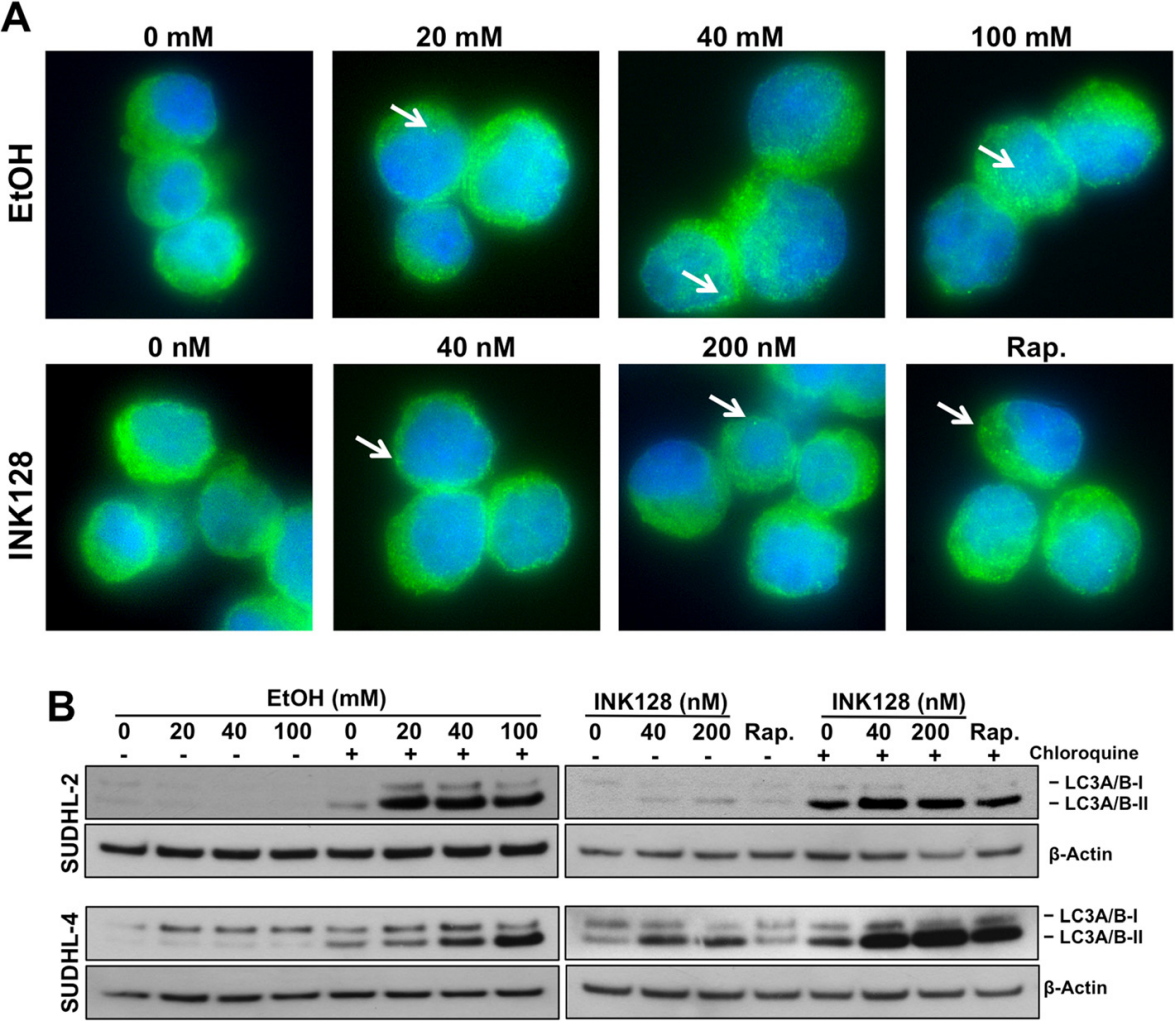
**EtOH and INK128 induce autophagy. (A)** SUDHL-2 cells were treated with 10 μM Chloroquine and with indicated doses of EtOH, INK128 or rapamycin for 6 h. LC3A positive autophagic puncta (arrows show examples) were visualized under fluorescence microscopy. Pictures were taken using a Nikon TE2000S **(B)** Cells were treated as in **(A)** with or without Chloroquine. Cell lysates were then analyzed for abundance of LC3 forms by western blotting. β-Actin was used as a loading control. The data are representative of three independent experiments.

**Figure 8:**
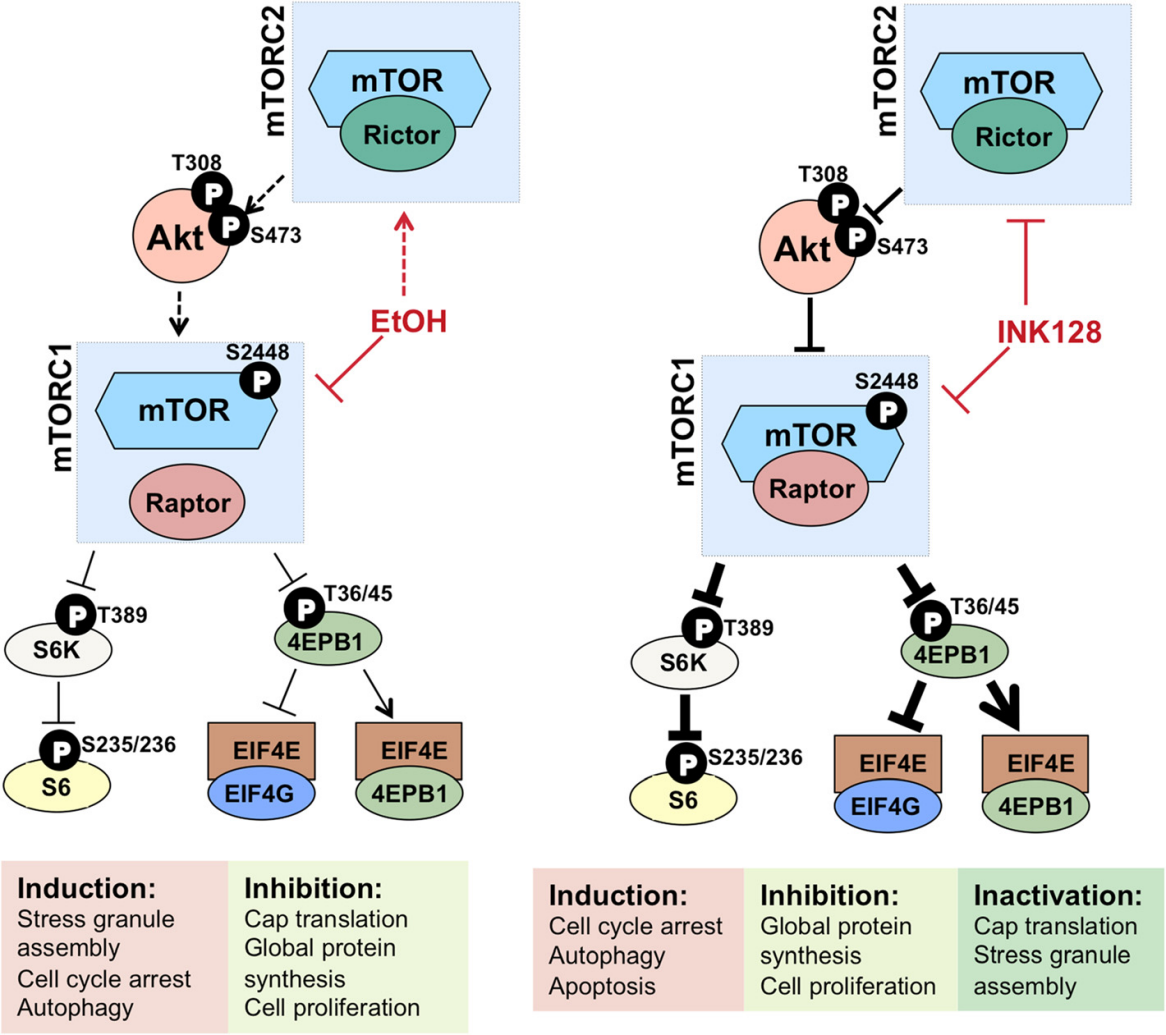
**Schematic of EtOH and INK128 impact on the mTOR pathway and cellular response.** Broken lines indicate no change or moderate increase.

Since mTORC1 regulates autophagy, and inhibition of mTOR has been shown to induce autophagic activity [36,37], we explored the potential of EtOH and INK128 to induce autophagy through the assessment of the autophagy marker microtubule-associated protein 1 light chain 3 (MAP1LC3 or LC3) in DLBCL. In the process of autophagy induction, LC3 is lipidated from the soluble form (LC3-I) to the autophagosome-associated form (LC3-II), which ultimately results in the formation of LC3-positive puncta (autophagosomes) within the cells [38]. Upon SUDHL-2 cell exposure to EtOH and INK128, as well as rapamycin, we observed increased autophagosome formation as visualized by LC3-positive puncta in immunofluorescent analysis (Figure 7A). Cells were also assessed by western blot to detect LC3-I conversion to LC3-II. We detected elevated levels of the autophagosome form (LC3-II) by western blotting in all doses and treatments (Figure 7B). In summary, the observed induction of autophagy by both EtOH and INK128 combined with the dose dependent induction of apoptosis by INK128 but not by EtOH treatment (Figure 6D) further corroborates a limited suppression of mTOR by EtOH, as opposed to the potent mTOR inactivation by INK128 with respect to cellular phenotype and subsequent cell fate (Figure 8).

## Discussion

mTOR is a central node in the regulation of critical cellular processes including protein synthesis, cell growth and metabolism, which are often aberrantly stimulated in many pathologies including cancer. To date, many pharmacological inhibitors have been discovered which inhibit mTOR signaling, from the classical allosteric inhibitor, rapamycin, to more recent active site inhibitors such as PP242, Torin1, AZD8055 and INK128, [7,26,27,39]. Nevertheless, due to the many molecular levels regulating mTOR signal transduction, compounded by cell type specificity, the exact mechanisms of regulation and the impact of current therapeutics remain underdeveloped [40]. While EtOH has been shown to inhibit the mTORC1 signaling pathway, these studies have mostly been performed on non-human tissue [12,13,22-25], there have been few studies on human malignancy [14]. In contrast to most cancer types, a growing number of population studies have shown an inverse correlation between EtOH consumption and incidence of hematological malignancy [17-21]. However, the mechanisms behind EtOH-based regulation of mTOR components, mTORC1 and mTORC2, and their functional consequences in hematological malignancies are poorly understood.

In this study, we chose the catalytic mTOR inhibitor INK128 to compare with EtOH for the purpose of elucidating convergent and divergent pathways between the two treatments, in the context of DLBCL. INK128 is a second-generation ATP-competitive mTOR inhibitor that binds the mTOR catalytic domain and selectively inhibits both mTORC1 and mTORC2, and is currently in clinical trials [26,41,42]. We have demonstrated that treatment of DLBCL with EtOH suppressed mTORC1 activity in a dose dependent manner with concomitant augmentation of AKT phosphorylation at both Thr308 and Ser473, whereas the dual mTORC1/2 inhibitor INK128 completely abrogated AKT phosphorylation at these sites. While Akt phosphorylation was increased with EtOH treatment there was no concomitant increase in mTOR S2448 phosphorylation. Given that intact mTORC1 complex is required for specific S2448 mTOR site phosphorylation [43] it is conceivable that EtOH-induced disruption of the raptor-mTOR complex (Figure 1C) prevented mTOR from Akt mediated phosphorylation. Our finding that EtOH decreased raptor-mTOR association while increasing rictor-mTOR complex formation provides strong evidence for EtOH not only having a suppressive effect on mTORC1 but also an activating role in the mTORC2 pathway. Breuleux et al. (2009) demonstrated that selective mTORC1 suppression by RAD001, or knock down of raptor, elicits increased AKT S473 phosphorylation, requires rictor and can be modulated by mTORC2 complex [44]. I addition, it was found that the rictor-mTOR complex directly phosphorylated AKT/PKB at Ser473, which facilitated Thr308 phosphorylation by PDK1 [5]. It is also possible that, EtOH may modulate the Akt-mTOR interaction, Akt catalytic activity or subcellular location. Activation of p70S6K has been shown to modulate the phosphorylation mTOR S2448 [45], so it may be that through the inhibition of p70S6K (via mTORC1 disruption) that AKT is no longer able to physically interact with and phosphorylate the S2448 site. Although previous research has demonstrated EtOH’s effect on mTOR to be both TSC1/2 and AMPK independent [14], EtOH may affect other unknown upstream kinases. Nearly full inhibition of mTORC1 and mTORC2 by INK128, however, did not cause changes in or slightly increased mTORC1/2 complex formation. This is likely due to a much stronger ablation of the negative feedback mechanisms that drive raptor and rictor dissociation, keeping complex activity balanced [46].

mTOR is known to modulate cap-dependent translation through the phosphorylation of the translation inhibitor 4E-BP1 and to subsequently prevent 4E-BP1 binding to eIF4E [6,47,48]. eIF4E is an essential component in initiating cap-dependent translation via its binding to 5’ 7-methyl-GTP cap structure on mRNAs, which stimulates formation of the cap-binding eIF4F complex through its interaction with the scaffolding protein eIF4G [49-51]. Since 4E-BP1 directly competes with eIF4G for the same eIF4E-binding site, mTORC1-induced 4E-BP1 phosphorylation results in more eIF4G binding to eIF4E, recruitment of the 40S ribosomal subunit to the 5′ cap and enhanced cap-dependent translation initiation [51-53]. Fournier et al. (2013) reported that mTORC1, through the phosphorylation of 4E-BP1 and consequent regulation of eIF4E-eIF4G association, facilitates SG formation [35]. We discovered that EtOH resulted in inhibition of mTORC1 phosphorylating 4E-BP1, consequently causing a moderate reduction in eIF4E-eIF4G complex assembly and promoting assembly of SG. We further demonstrated that INK128-induced mTOR inhibition almost completely abolished the mTORC1-eIF4E pathway, leading to impaired SG formation. This result is consistent with the recent data showing that pharmacological inactivation of the mTOR pathway by PP242 resulted in failure of SG assembly [35]. The authors demonstrated that PP242-induced hypo-phosphorylation of 4E-BP1 and abrogation of eIF4E-eIF4G interaction by 4E-BP1 prevents eIF4E-mediated SG formation. We thus postulate that, similar to PP242, a possible mechanism by which INK128 impairs SG formation is the complete disruption of eIF4E-eIF4G complex by 4E-BP1, following treatment. Given that eIF4E-eIF4G complexes might be essential for assembly of SG under mild stress conditions [35], we speculate that the moderate decrease in mTOR signaling caused by EtOH and consequent eIF4E-eIF4G partial association allow for and promote SG formation. However, future experiments are needed to establish the role mTOR signaling in SG formation.

Using microarray analysis of actively translated polysomal mRNA, we found that EtOH affected the translation of fewer mRNAs than INK128. Intriguingly, almost 80% of the genes did not overlap between the two treatments, indicating that EtOH and INK128 exposure distinctly modulate the DLBCL translatome while confirming a previous study which detailed distinct translational profiles with different mTOR inhibitors [48]. Despite finding that most of the genes were unique to each treatment, many of those genes differentially controlled by EtOH and INK128 are involved in similar pathways such as AKT/mTOR signaling, protein synthesis, cell cycle, proliferation and apoptosis. It has been previously reported that some of the mRNAs involved in protein synthesis and translationally controlled by mTOR may contain 5’ terminal oligopyrimidine (5’TOP) or TOP-like pyrimidine-rich translational element (PRTE) motifs within their 5’ untranslated regions (5’ UTRs) [26,54-56]. We found that 21.4% of the INK128 regulated genes possess a PRTE-like motif, however instead of thymidine enrichment in the 6^th^ position, thymidine appears in the 5^th^ (Additional file 8: Figure S6). In the group of genes whose expression was altered by EtOH treatment, no statistically significant motifs were found. However, future studies are needed to determine the regulatory mechanism for these sequences and their involvement in the mTOR drug response.

The fact that EtOH treatment less markedly affected gene expression is consistent with EtOH incompletely interfering with the activity of mTORC1 when compared to the efficient inhibition of mTORC1-dependent eIF4E activation by INK128. Our findings further establish that although the end result of mTOR inhibition is a global decrease in protein synthesis, differential inhibition of mTOR signal transduction may distinctly modulate translation of specific subsets of mRNAs that control the key cellular processes responsible for cell fate.

Despite its modest effect on translation, treatment with EtOH resulted in a similar cellular phenotype to INK128, albeit weaker, by inhibiting cell cycle progression and proliferation while promoting the induction of autophagy. While active mTORC1 promotes cell growth, it also negatively regulates autophagy through the phosphorylation-driven repression of the ULK1/Atg13/FIP200 kinase complex. Autophagy involves the cellular degradation of unnecessary or dysfunctional cellular components via lysosomes. This degradative pathway promotes cell survival during starvation or other stressors by maintaining cellular energy levels. With respect to cancer, autophagy is often associated with a pro-survival phenotype, whereas in other cases it can promote cell death [57]. It is therefore interesting that INK128, although responsible for the induction of autophagy, also induced cell apoptosis, suggesting that while EtOH decreases DLBCL cell viability, full inhibition of mTOR cannot be salvaged by autophagy. Finally, it is important to note that in contrast to the specific activity of INK128, EtOH can exert many pleiotropic effects in addition to the suppression of mTOR signaling and protein synthesis. Ethanol exposure has been shown to produce reactive oxygen species (ROS) and elicit an oxidative stress response [22]. Ethanol has also been shown to induce ER stress-mediated neuronal cell death thus limiting the interpretation of our data [23]. It remains to be determined if other pleiotropic effects of EtOH participate in, or can account for, its overall influence on hematological malignancies.

## Conclusions

In summary, we provide evidence that EtOH partially inhibits mTOR activity compared to INK128’s complete suppression of mTOR, resulting in differential regulation of downstream mTOR targets and consequent differences in cellular responses and cell fate (Figure 8). Although INK128 can fully inhibit the activity of mTOR, we have shown through a comprehensive investigation of signal transduction, translatome analysis, and phenotypic observation that when comparing EtOH treatment to an equipotent dose of INK128, significant differences are seen that may account for a decreased incidence of hematological malignancy in alcohol consumers. These findings additionally support a model for mTOR functioning as a ‘master’ upstream regulator of a functionally related subset of mRNAs and further reveals the complexity of its control.

## Materials and methods

### Cell culture and treatments

The collection of tissues was determined by our University of Maryland Medical School IRB to be Not Human Subjects Research, as no identifying information was collected from subjects, therefore no written informed consent was required or collected. SUDHL-2 and SUDHL-4 diffuse large B-cell lymphoma (DLBCL) cell lines (ATCC) were cultured in RPMI Medium 1640 (Gibco BRL) supplemented with 10% fetal bovine serum (FBS) at 37°C in 5% CO_2,_ as previously described [58]. Cells were treated with EtOH at concentrations of 20, 40 or 100 mM for 24 h, unless differently specified, and cultured in sealed flasks to maintain EtOH concentrations in the culture medium, as previously described [13,22,25]. Cells were treated with INK128 (Selleckchem) dissolved in dimethyl sulfoxide (DMSO). Rapamycin (Sigma) was used at a concentration of 20 nM. Vehicle controls were treated with 0.01% DMSO.

### Primary human tonsillar B-cell isolation and lymphoblastoid cell line generation

Primary human tonsillar B-cells were provided by the University of Maryland Greenebaum Cancer Center Pathology Biorepository and Research Core from routine tonsillectomies in accordance with the guidelines of the University of Maryland Medical School Institutional Review Board and conform to the Declaration of Helsinki. Tonsils were minced on ice and lymphocyte populations were separated by Ficoll, washed in Hank’s Balanced Salt Solution (HBSS) and stained with CD19-APC (BD Bioscience), CD38-FITC (BD Bioscience) and IgM-PE (eBioscience) in HBSS containing 5% FBS. Cells were then sorted through a 70 μM nozzle on a FACSAria II (BD Bioscience) cell sorter to a purity of greater than 99%. Both CD19+/CD38+/IgM- (germinal center) and CD19+/CD38-/IgM+ (naive) B-cells were then cultured in RPMI containing 10% FBS, 1% Pen/Strep and 1% amphotericin B while in the presence of Epstein-Barr virus (EBV) (ATCC VR-1492) for 6 weeks until outgrowth was observed.

### Western blotting and co-immunoprecipitation assay

For western blot analysis, protein lysates (10–50 μg) were size-separated by SDS-PAGE (Invitrogen), transferred onto PVDF membranes and probed with the indicated antibodies. Blots were probed with polyclonal rabbit antibodies recognizing p-mTOR (Ser 2448), mTOR, p-p70 S6 kinase (Thr 389), p70 S6 kinase, p-RPS6 (Ser 235/236), RPS6, p-4E-BP1 (Thr 36/45), 4E-BP1, p-AKT (Ser 473), p-AKT (Thr 308), Lyn, eIF4G, Caspase-3 and LC3A/B (Cell Signaling Technology), eIF4E, PARP-1, YWHAZ (Santa Cruz Biotechnology), Raptor, Rictor, MARS (Bethyl Laboratories), AKT (Upstate), polyclonal goat anti-FKBP11 antibody from Santa Cruz Biotechnology, or monoclonal mouse antibodies recognizing β-Actin (Abcam), CDC25A and TLC1A (Santa Cruz Biotechnology). After incubation with appropriate secondary antibodies, signals were detected by enhanced chemiluminescence (Pierce). For co-immunoprecipitation assays, IgG or mTOR antibodies (Cell Signaling Technology) were added to 0.2 mg protein lysates and incubated overnight at 4°C. Following incubation (1 h, 4°C) with Protein A-Sepharose beads (Invitrogen) complexes were washed three times with NT2 buffer (50 mM Tris pH 7.4, 150 mM NaCl, 1 mM MgCl_2_, 0.05% NP-40) and analyzed by western blotting.

### Polyribosome fractionation

Cells were lysed in cytoplasmic lysis buffer (5 mM Tris, 2.5 mM MgCl_2_, 1.5 mM KCl, 1% Triton X-100, 0.5% Sodium Deoxycholate and 2 mM DTT), loaded on 10 - 50% linear sucrose gradients and fractionated as previously described [58,59]. The RNA in each fraction was monitored by optical density measurement (A254) and eleven fractions were collected with a fraction collector (Brandel). The RNA from each fraction was isolated by Trizol (Invitrogen) and used for RT-qPCR analysis. RNA from high molecular weight polysomal fractions, with actively translated mRNAs (fractions 9–11), were pooled and used for microarray analysis.

### Analysis of newly translated protein

Analysis of *de novo* translation was performed as reported previously [58]. Briefly, after treatments, cells were incubated with L-[^35^S]methionine and L-[^35^S]cysteine (Easy Tag EXPRESS; NEN/Perkin-Elmer) for 20 min and radiolabel incorporation was monitored by resolving cell lysates on SDS-PAGE followed by transfer onto PVDF membranes and visualization with a PhosphorImager (GE Healthcare).

### m7GTP pull-down and luciferase assay

7-methyl-GTP cap analog pull-down was carried out as previously described [58]. Shortly, 500 ug of total cell lysates were incubated with the 7-methyl-GTP cap analog bound to Sepharose beads (Jena Bioscience), washed, and the cap-bound protein complex was eluted and analyzed by western blotting.

### Immunofluorescence

Cells were fixed in 1% paraformaldehyde and permeabilized in PBS with 0.5% Triton X-100. After washing with 0.1% PBST, cells were incubated in IF blocking buffer (3% BSA, 0.1% Tween-20 in PBS) for 1 h at RT. Cells were then incubated overnight at 4°C with mouse anti-G3BP1, goat anti-TIAR (Santa Cruz Biotechnology), or sheep anti-LC3A (Abcam) antibodies in blocking buffer (1:200) and washed with PBS + 0.1% Tween-20. They were further incubated for 1 h at RT with the appropriate secondary goat anti-mouse Alexa Fluor 568, donkey anti-sheep Alexa Fluor 488 and donkey anti-goat Alexa Fluor 488 secondary antibodies (Molecular Probes; 1:200 dilution) and washed with PBS + 0.1% Tween-20. The stained cells were seeded on slides and mounted using ProLong Gold mounting medium with DAPI (Invitrogen). Photos were taken using a fluorescence microscope (Nikon TE2000S).

### Analysis of cell cycle, apoptosis and autophagy

Cells were fixed with 70% EtOH, washed with PBS, stained using PI/RNase staining buffer (BD Biosciences) and analyzed for cell cycle with a flow cytometer. Apoptosis was analyzed by flow cytometry with the PI/Annexin V staining kit (BD Biosciences). Autophagy was analyzed by treatment with 10 μM chloroquine diphosphate (Sigma) and with either EtOH, INK128 or rapamycin for 6 h. Appearance of LC3A positive autophagic puncta was assessed by immunofluorescence microscopy and, indicative of autophagic activity, conversion of LC3-I to LC3-II was monitored by western blotting.

### Microarray data analysis

Microarray and data analysis was performed as previously described [58]. Briefly, RNA isolated from sucrose fractions was labeled with Illumina TotalPrep RNA Amplification Kit (Ambion; Austin, TX) and analyzed using human HT-12 v1.0 gene expression BeadChips containing 48,000 RefSeq transcripts (Illumina, San Diego, CA). Microarray data were filtered by the detection p-value ≤ 0.02, normalized by Z-score transformation, tested for significant differences in signal intensity and analyzed for sample quality. Genes were considered significantly changed after calculating Z-ratio, indicating fold difference (Z > 1.5 or < −1.5), false discovery rate (fdr ≤ 0.3) and p <0.05. Differentially expressed genes were analyzed by Ingenuity Pathways Analysis (IPA) to identify the top functional networks and Gene Ontology (GO) analysis to identify key biological categories that were significantly changed in EtOH- or INK28-treated versus untreated control cells. See GEO database www.ncbi.nlm.nih.gov/geo/query/acc.cgi?acc=GSE62790 for complete microarray data.

### RT-qPCR analysis

Total and polysomal RNA were reverse transcribed with the iScript cDNA synthesis kit (Quanta Biosciences), and qPCR analysis was carried out using iQ SYBR Green Supermix (Quanta Biosciences) on a BioRad CFXConnect instrument. Oligonucleotides used for detection of specific mRNAs in each fraction from sucrose gradients are as follows: CTGGCTAAAGCTGGTGAAGG and TGGGTCTATTGGCCTTTCTG for FKBP11 mRNA, CCGCTGGTTTAACATTTCGT and TCAGCAACTGCTGGAAAATG for MARS mRNA, CATGGACTCCAGGAGGGTAA and TCACAGGTGACTGGGGTGTA for CDC25A mRNA, TATGACCCCCACCCAGATAG and ACTAAGCGCCAGAAACTGGA for TCL1A mRNA, GGAGGAGCCCATTTACATCA and ATGTATGCCATTCCCTCTGC for LYN mRNA, AGACGGAAGGTGCTGAGAAA and GAAGCATTGGGGATCAAGAA for YWHAZ mRNA and, CGGAGTCAACGGATTTGGTCGTAT and AGCCTTCTCCATGGTGGTGAAGAC for GAPDH mRNA.

## Additional files

Additional file 1: Figure S1. DLBCL cells were treated with EtOH for 24 h or with INK128 for 3 h at the indicated concentrations. Cell lysates were analyzed by western blotting. β-Actin served as a loading control. The data are representative of three independent experiments. Additional file 2: Figure S2. EtOH and INK128 influence on mTORC1/2 activity in B-lymphoblastoid cell lines. GCB or naïve B-cells were treated with EtOH for 24 h, with INK128 for 3 h at the concentrations as indicated, or 20 nM rapamycin (Rap.) for 3 h and the phosphorylation states and total levels of indicated proteins were detected by western blotting. Additional file 3: Table S1. Genes with the most altered translation induced by EtOH and INK128 in DLBCL cells. Additional file 4: Table S2. The top five functional networks identified by Ingenuity Pathways Analysis (IPA) from the genes translationally regulated by EtOH or INK128. Additional file 5: Figure S3. Functional categories of polysome-associated mRNAs in EtOH (20 mM) and INK128 (40 nM) treated DLBCL cells. Heat map represents the top annotations with greatest representation of altered genes. Additional file 6: Figure S4. Representative picture of flow cytometry cell cycle analysis performed 48 h after SUDHL-2 cells treated with indicated doses of EtOH, INK128 or 20 nM rapamycin (Rap.). Additional file 7: Figure S5. Representative picture of flow cytometry analysis of Annexin V/PI staining in SUDHL-2 cells 72 h after treatment with indicated doses of EtOH, INK128 or rapamycin. Additional file 8: Figure S6. Pyrimidine Rich Translational Element (PRTE) motif found within the 5’ UTRs of 21.4% of INK128 responsive translationally regulated mRNAs by MEME (Multiple EM for Motif Elucidation).

## Abbreviations

*4E*-*BP1*: Eukaryotic translation initiation factor 4E binding protein 1
*5*’ *UTR* 5’: Untranslated region
*5*’*TOP* 5’: Terminal oligopyrimidine
*AKT*: Protein kinase B, a serine/threonine specific protein kinase
*DLBCL*: Diffuse large B-cell lymphoma
*eIF4E*: Eukaryotic translation initiation factor 4E
*EtOH*: Ethanol
*GO*: Gene Ontology analysis
*IPA*: Ingenuity Pathway Analysis
*m7GTP*: 7-methyl guanosine 5’-triphosphate
*MAP1LC3 or LC3*: Microtubule-associated protein 1 light chain 3
MEME: Multiple EM for Motif Elucidation
*mRNP*: Messenger ribonucleo-protein
*mTOR*: Mechanistic target of rapamycin, or mammalian target of rapamycin
*PRTE*: Pyrimidine-rich translational element
*RPS6*: Ribosomal protein S6
*S6K1* or *p70S6K*: S6 kinase 1
*SG*: Stress granule
*SGK*-*1*: Serum/glucocorticoid-regulated kinase 1
*TORKinib*: TOR kinase domain inhibitor
