# Supplementary figures and images for: Distinct inhibitory effects on mTOR signaling by ethanol and INK128 in diffuse large B-cell lymphoma
Source: Cell Commun Signal. 2015 Mar 1;13:15. doi: 10.1186/s12964-015-0091-0 (PMC4350884; doi:10.1186/s12964-015-0091-0)

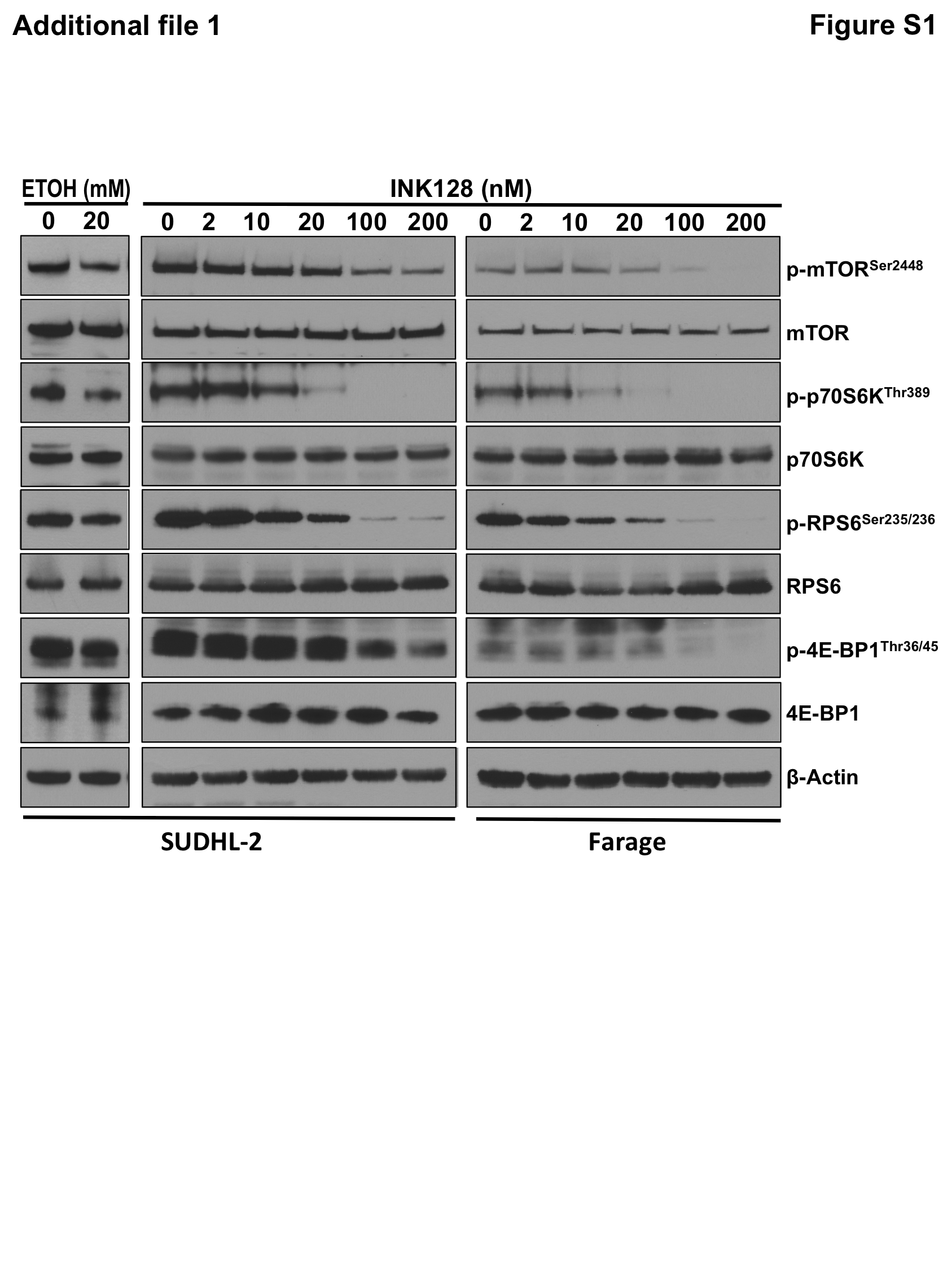

Supplement: Additional file 1: Figure S1. — DLBCL cells were treated with EtOH for 24 h or with INK128 for 3 h at the indicated concentrations. Cell lysates were analyzed by western blotting. β-Actin served as a loading control. The data are representative of three independent experiments. [file 12964_2015_91_MOESM1_ESM.tiff]

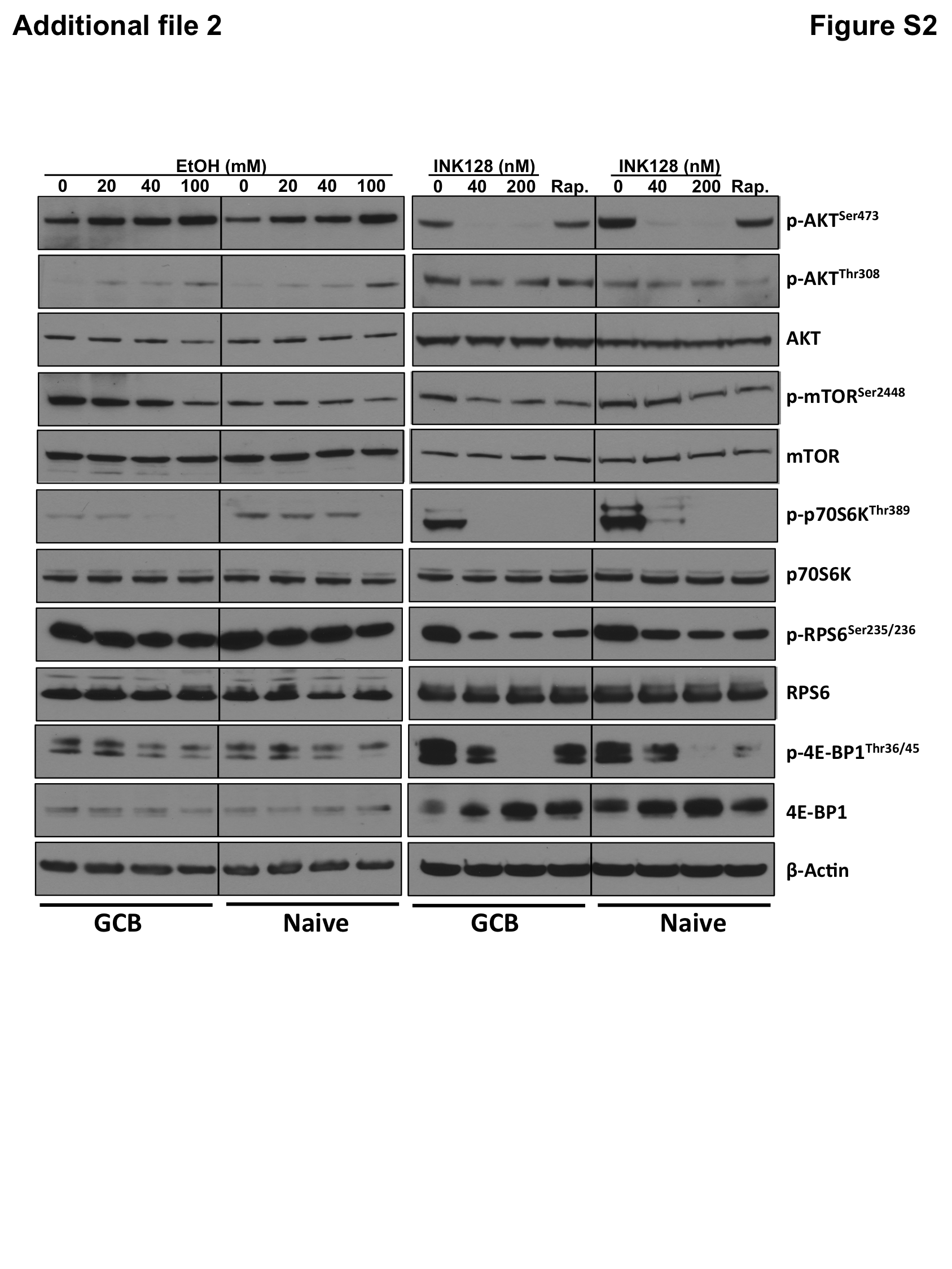

Supplement: Additional file 2: Figure S2. — EtOH and INK128 influence on mTORC1/2 activity in B-lymphoblastoid cell lines. GCB or naïve B-cells were treated with EtOH for 24 h, with INK128 for 3 h at the concentrations as indicated, or 20 nM rapamycin (Rap.) for 3 h and the phosphorylation states and total levels of indicated proteins were detected by western blotting. [file 12964_2015_91_MOESM2_ESM.tiff]

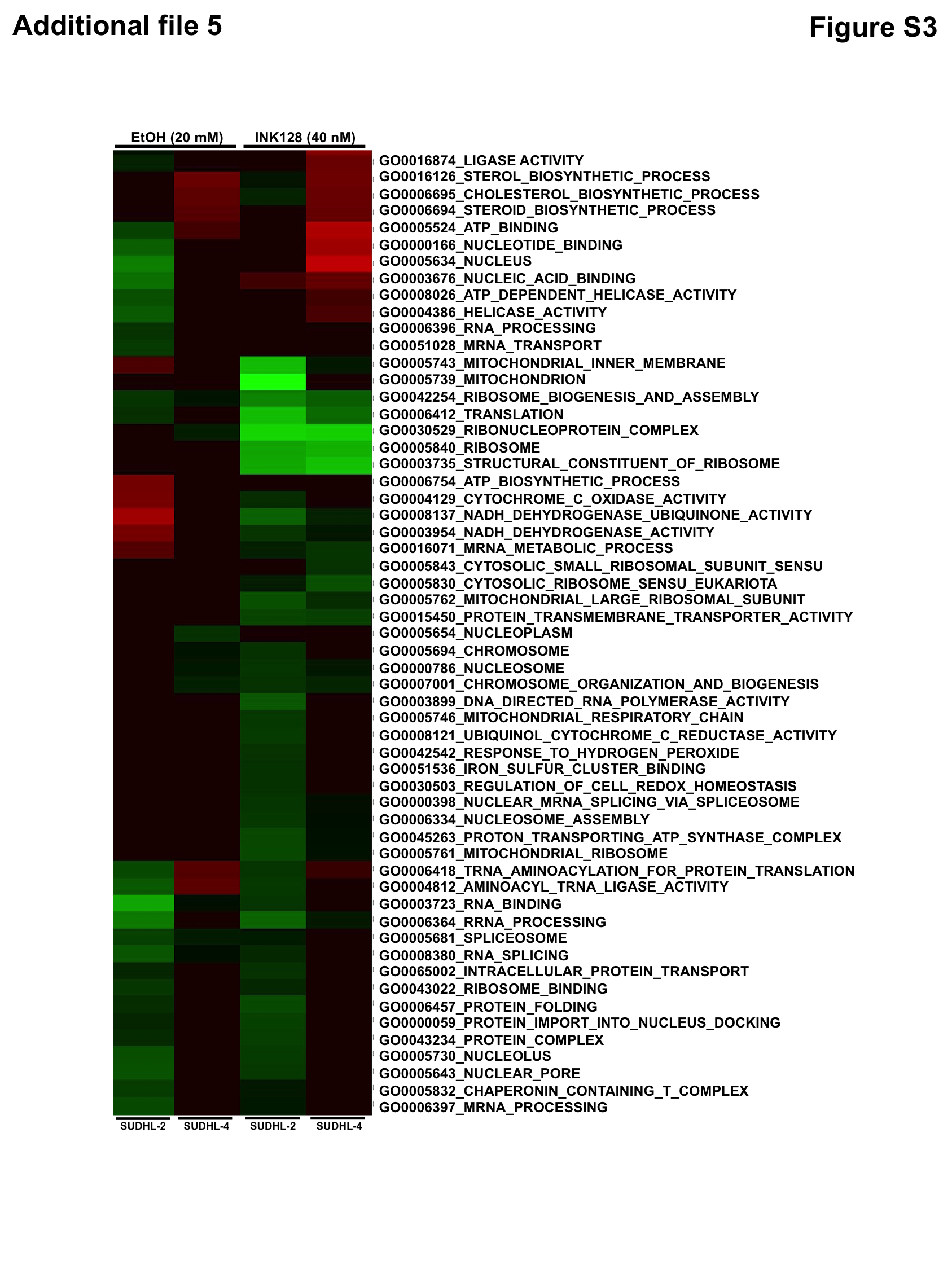

Supplement: Additional file 5: Figure S3. — Functional categories of polysome-associated mRNAs in EtOH (20 mM) and INK128 (40 nM) treated DLBCL cells. Heat map represents the top annotations with greatest representation of altered genes. [file 12964_2015_91_MOESM5_ESM.tiff]

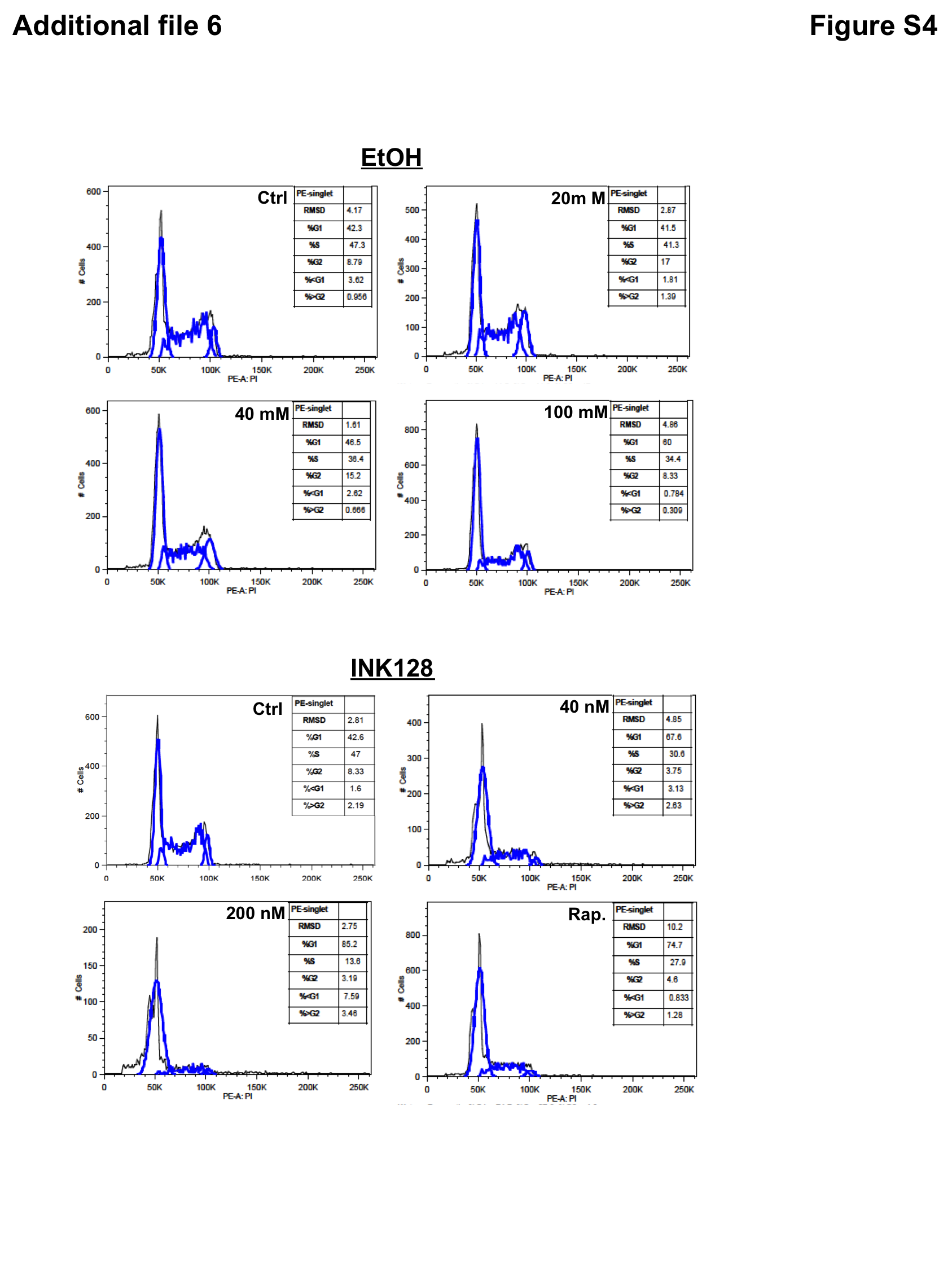

Supplement: Additional file 6: Figure S4. — Representative picture of flow cytometry cell cycle analysis performed 48 h after SUDHL-2 cells treated with indicated doses of EtOH, INK128 or 20 nM rapamycin (Rap.). [file 12964_2015_91_MOESM6_ESM.tiff]

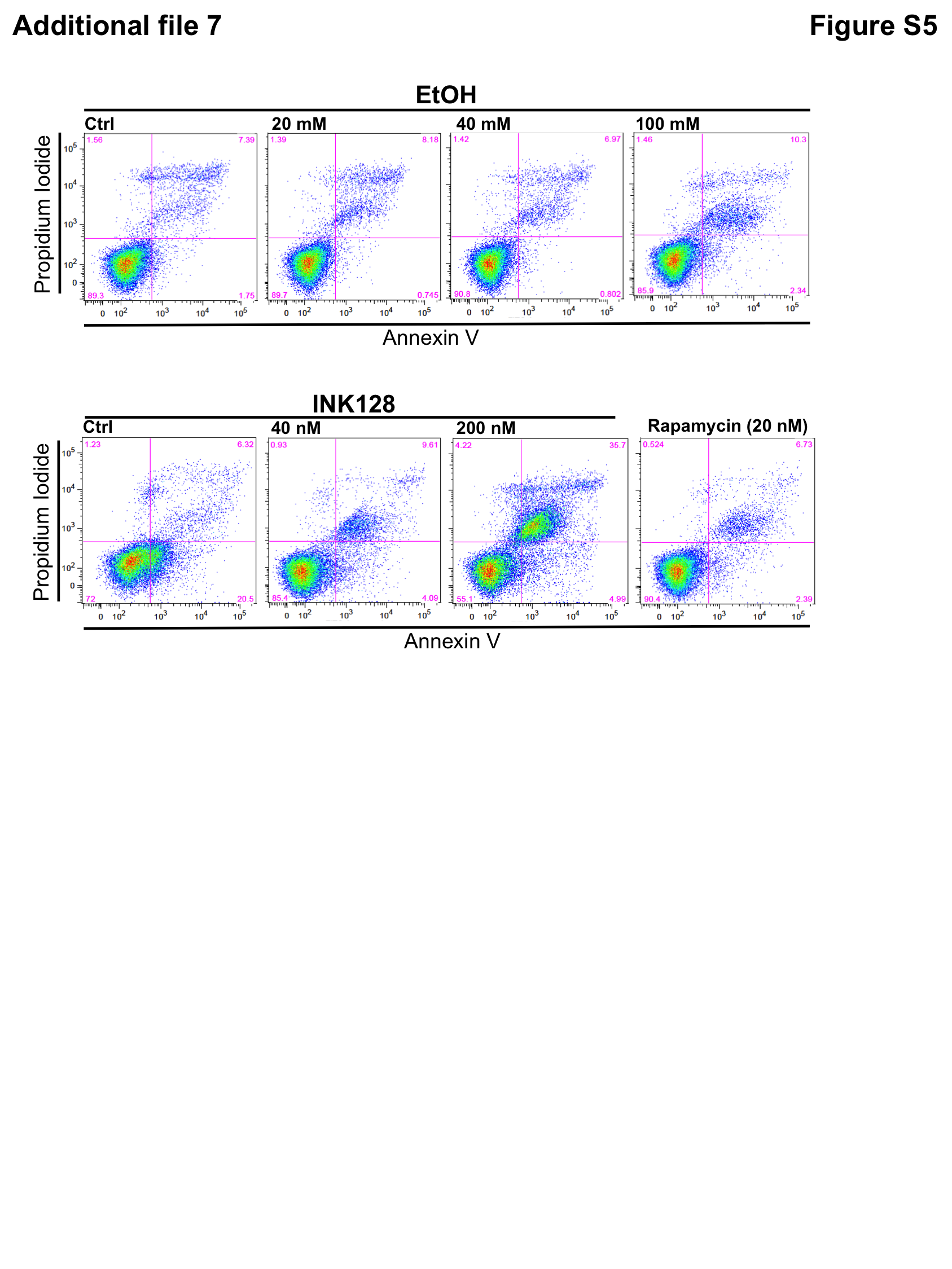

Supplement: Additional file 7: Figure S5. — Representative picture of flow cytometry analysis of Annexin V/PI staining in SUDHL-2 cells 72 h after treatment with indicated doses of EtOH, INK128 or rapamycin. [file 12964_2015_91_MOESM7_ESM.tiff]

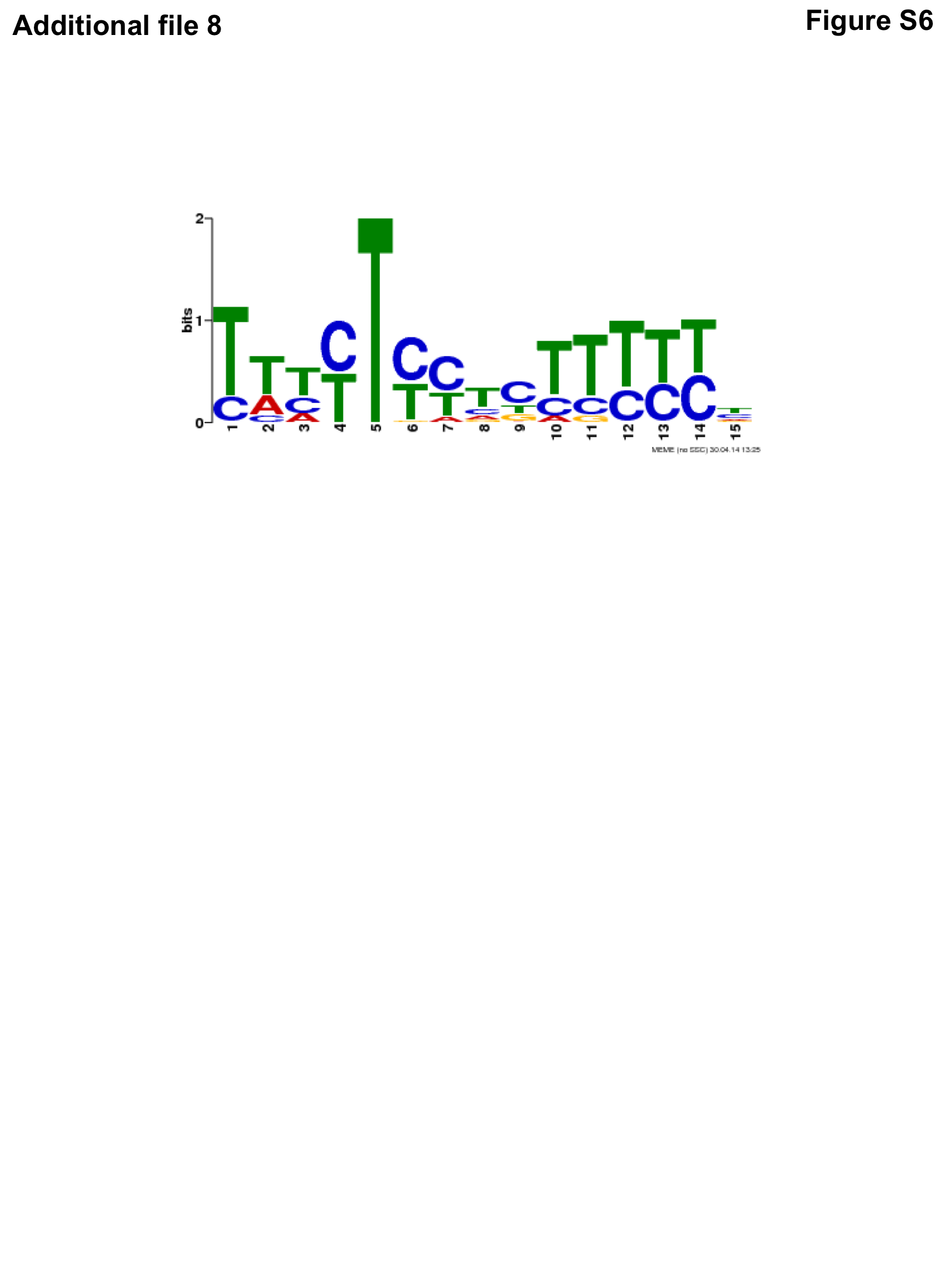

Supplement: Additional file 8: Figure S6. — Pyrimidine Rich Translational Element (PRTE) motif found within the 5’ UTRs of 21.4% of INK128 responsive translationally regulated mRNAs by MEME (Multiple EM for Motif Elucidation). [file 12964_2015_91_MOESM8_ESM.tiff]
