# Supplementary material for: Distinct inhibitory effects on mTOR signaling by ethanol and INK128 in diffuse large B-cell lymphoma
Source: Cell Commun Signal. 2015 Mar 1;13:15. doi: 10.1186/s12964-015-0091-0 (PMC4350884; doi:10.1186/s12964-015-0091-0)
Supplement: Additional file 3: Table S1. — Genes with the most altered translation induced by EtOH and INK128 in DLBCL cells. [file 12964_2015_91_MOESM3_ESM.docx]

**Table S1. Genes with the most altered translation induced by EtOH and INK128 in DLBCL cells**

| Gene Name | EtOH (SUDHL-2)  z-ratio | EtOH (SUDHL-4)  z-ratio | INK128 (SUDHL-2)  z-ratio | INK128 (SUDHL-4)  z-ratio |
| --- | --- | --- | --- | --- |
| LOC100131866 | -8.83 | -3.44 | -1.82 | 2.29 |
| RPL8 | -8.08 | -3.84 | -1.13 | -0.95 |
| LOC653383 | -7.84 | -1.73 | -1.82 | 0.11 |
| LTBP3 | -7.82 | -1.51 | 2.07 | 0.55 |
| RPLP0 | -5.655 | -3.435 | -1.41 | 0.69 |
| LOC100129267 | -5.65 | -4.25 | 0.74 | -1.63 |
| LOC100134648 | -5.41 | -3.84 | -0.75 | -0.49 |
| NUTF2 | -2.17 | -4.91 | 0.88 | -1.25 |
| YWHAZ | -4.63 | -2.61 | -0.78 | 0.56 |
| SNRPD3 | -3.3 | -4.59 | 0.71 | 0.62 |
| C19orf48 | -3.665 | -4.41 | 0.805 | -0.495 |
| LOC651816 | -3.89 | -4.37 | 2.95 | -0.69 |
| TMEM160 | -3.99 | -3.64 | 3.39 | -0.82 |
| LOC728873 | -3.1 | -3.4 | 3.97 | -1.46 |
| RBM14 | 0.82 | -0.65 | -5.42 | -2.79 |
| CCDC86 | 0.4 | -1.42 | -3.81 | -5.18 |
| HSPA5 | -0.08 | -0.95 | -5.15 | -3.77 |
| LOC100130769 | 0.17 | -0.92 | -2.16 | -5.11 |
| CHAC2 | -0.1 | -0.64 | -4.99 | -4.16 |
| CDC25A | 0.84 | -0.01 | -2.88 | -4.82 |
| RRS1 | -0.46 | -0.14 | -4.78 | -3.31 |
| LOC645630 | -0.64 | -0.43 | -4.63 | -2.27 |
| LOC646949 | 0.12 | 0.26 | -4.53 | -1.54 |
| SRXN1 | 0.39 | 0.86 | -2.38 | -4.52 |
| LOC442232 | 1.25 | -1.76 | -4.46 | -1.74 |
| LOC100131971 | 0.59 | 0.27 | -1.71 | -4.44 |
| FKBP4 | 0.88 | -0.84 | -4.4 | -3.96 |
| LOC641992 | -1.01 | 2.49 | -4.38 | -2.34 |
| LOC100129657 | 0.18 | 2.02 | -4.35 | -2.46 |
| LOC729608 | -0.59 | 0.1 | -4.33 | -2.89 |
| DPH5 | -0.785 | -0.88 | -4.215 | -2.03 |
| LOC391075 | -0.31 | -1.23 | -4.18 | -3.01 |
| CDC42 | -1.38 | -0.56 | -4.14 | -3.84 |
| MARS | 1.36 | 0.62 | -3.19 | -4.13 |
| MARS2 | -0.25 | 1.19 | -4.12 | -2.27 |
| IER3 | 0.39 | 0.62 | -4.11 | -3.3 |
| GRPEL1 | -0.56 | -0.23 | -4.07 | -2.64 |
| RPF2 | -0.03 | 0.38 | -4.07 | -3.07 |
| FKBP11 | -0.57 | -1.4 | -3.24 | -4.06 |
| LOC100133697 | 0.59 | 0.09 | -4.04 | -2.19 |
| LOC728791 | 1.13 | 0.34 | -4.03 | -2.29 |
| LOC652595 | -0.02 | 0.24 | -4.01 | -3.86 |
| ATF5 | 0.16 | -0.75 | -3.98 | -2.28 |
| SYT17 | 0.92 | 1.28 | 3.6 | 7.6 |
| SNORA12 | -0.28 | -2.23 | 3.25 | 6.36 |
| HPS3 | -1.72 | 2.36 | 6.32 | 1.75 |
| NCF1C | 0.52 | 0.22 | 1.96 | 6.06 |
| RASGRP3 | 1.09 | 1.52 | 4.84 | 6 |
| HBP1 | 0.165 | 1.605 | 3.685 | 5.78 |
| TCL1A | 1.46 | 0.16 | 5.34 | 5.67 |
| LRMP | 1.12 | -0.94 | 5.64 | 1.81 |
| LOC401152 | -0.31 | 0.28 | 5.45 | 2.32 |
| TSPAN31 | 1.03 | 1.3 | 2.66 | 5.28 |
| C9orf103 | -0.24 | 0.63 | 5.25 | 2.71 |
| LOC644914 | 1.34 | -1.2 | 5.16 | 2.44 |
| PNPLA7 | -0.27 | 0.7 | 3.95 | 5.08 |
| TLR6 | 0.21 | 1.47 | 3.54 | 5.02 |
| C11orf67 | 0.14 | -0.79 | 4.93 | 2.23 |
| HLA-DMB | 1.02 | 0.42 | 3.31 | 4.77 |
| C4orf34 | 0.765 | -0.525 | 2.24 | 4.76 |
| CD27 | 0.93 | 0.62 | 4.61 | 4.76 |
| SPSB3 | 0.25 | -0.43 | 4.7 | 3.79 |
| WDR40A | 1.1 | -0.46 | 2.25 | 4.69 |
| CYBASC3 | 0.84 | 1.54 | 4.13 | 4.62 |
| TCL1B | 0.32 | -0.45 | 4.59 | 4.61 |
| C3orf37 | 0.44 | -0.59 | 2.335 | 4.505 |
| LOC100129034 | 0.17 | 0.91 | 4.47 | 3.22 |
| FCRLA | 0.72 | -0.51 | 4.46 | 2.23 |
| C8orf40 | 1.15 | 1.65 | 4.45 | 2.05 |
| PIM2 | -0.11 | 1.63 | 4.4 | 2.47 |
| SPATA20 | 1.16 | 0.27 | 3.62 | 4.37 |
| FLOT2 | -1.13 | -1.46 | 4.32 | 1.64 |
| KCNMB3 | -0.1 | 1.79 | 2.23 | 4.32 |
| ATP8A1 | 0.58 | 0.8 | 4.27 | 2.52 |
| TMEM149 | -0.24 | 0.87 | 2.08 | 4.18 |
| TOPBP1 | -0.08 | 1.74 | 4.18 | 2 |
| COMMD6 | 1.3 | -0.08 | 4.14 | 1.56 |
| GMFG | 0.13 | -0.49 | 3.17 | 4.14 |
| C1orf97 | 0.24 | 0.25 | 4.135 | 2.63 |
| RFTN1 | 0.09 | 0.04 | 1.72 | 4.13 |
| C5 | 0.29 | 0.28 | 2.78 | 4.12 |
| C16orf75 | 1.04 | 1.28 | 4.09 | 3.23 |
| CTPS2 | 0.75 | 0.605 | 2.895 | 4.035 |
| LYN | 0.07 | -0.17 | 3.57 | 4.02 |
| FAM100B | -1.15 | 1.08 | 3.99 | 1.64 |
| C6orf223 | 3.37 | 5.31 | 0.57 | -0.52 |
| ITGB7 | 2.2 | 4.34 | -1.51 | -0.02 |
| ANKRD37 | 2.53 | 4.03 | 0.94 | 0.42 |
| NCDN | 1.85 | 3.99 | 2.41 | -0.41 |
| HLA-DOB | 2.3 | 1.92 | 4.19 | 5.25 |
| SLAMF6 | 0.445 | 2.155 | 1.5 | 5.025 |
| HLA-DMA | 2.53 | 1.76 | 2.66 | 4.02 |
| NIN | 0.785 | 2.755 | 3.9475 | 1.4425 |
