# Supplementary material for: Distinct inhibitory effects on mTOR signaling by ethanol and INK128 in diffuse large B-cell lymphoma
Source: Cell Commun Signal. 2015 Mar 1;13:15. doi: 10.1186/s12964-015-0091-0 (PMC4350884; doi:10.1186/s12964-015-0091-0)
Supplement: Additional file 4: Table S2. — The top five functional networks identified by Ingenuity Pathways Analysis (IPA) from the genes translationally regulated by EtOH or INK128. [file 12964_2015_91_MOESM4_ESM.doc]

**Table S2 The top five functional networks identified by Ingenuity Pathways Analysis (IPA) from the genes translationally regulated by EtOH or INK128**

|  | **ID** | **Associated Network Functions** | **Score** |
| --- | --- | --- | --- |
| SUDHL-2 EtOH | 1 | Cell Death and Survival, Hematological System Development and Function, Tissue Morphology | 124 |
|  | 2 | Cell Death and Survival, Hematological System Development and Function, Tissue Morphology | 13 |
|  | 3 | Dermatological Diseases and Conditions, Immunological Disease, Cardiovascular Disease | 0 |
|  | 4 | Cellular Compromise, Connective Tissue Development and Function, Embryonic Development | 0 |
|  | 5 | Cellular Assembly and Organization, Cellular Development, Cellular Function and Maintenance | 0 |
| SUDHL-4 EtOH | 1 | Hematological System Development and Function, Tissue Morphology, Cellular Development | 57 |
|  | 2 | Cellular Development, Cellular Growth and Proliferation, Hematological System Development and Function | 31 |
|  | 3 | Cellular Assembly and Organization, DNA Replication, Recombination, and Repair, Cell Cycle | 0 |
|  | 4 | Cell-To-Cell Signaling and Interaction, Cellular Function and Maintenance, Hematological System Development and Function | 0 |
|  | 5 | Cellular Development, Hematological System Development and Function, Hematopoiesis | 0 |
| SUDHL-2 INK128 | 1 | Hematological System Development and Function, Tissue Morphology, Cell Death and Survival | 52 |
|  | 2 | Cell Death and Survival, Cellular Development, Cellular Growth and Proliferation | 22 |
|  | 3 | Cellular Development, Hematological System Development and Function, Hematopoiesis | 1 |
|  | 4 | Dermatological Diseases and Conditions, Developmental Disorder, Hereditary Disorder | 1 |
|  | 5 | Nervous System Development and Function, Organ Morphology, Tissue Morphology | 1 |
| SUDHL-4 INK128 | 1 | Hematological System Development and Function, Tissue Morphology, Cellular Development | 100 |
|  | 2 | Cell Death and Survival, Cellular Development, Cellular Growth and Proliferation | 31 |
|  | 3 | Hematological System Development and Function, Tissue Morphology, Cellular Development | 10 |
|  | 4 | Cellular Development, Cellular Growth and Proliferation, Dermatological Diseases and Conditions | 1 |
|  | 5 | Cell-To-Cell Signaling and Interaction, Cellular Movement, Hematological System Development and Function | 1 |
